# Supplementary material for: A semi-automatic approach to study population dynamics based on population pyramids
Source: MethodsX. 2025 Sep 2;15:103591. doi: 10.1016/j.mex.2025.103591 (PMC12450563; doi:10.1016/j.mex.2025.103591)
Supplement: Supplementary file 1 [file mmc1.docx]

**Supplementary Material**

**A semi-automatic approach to study population dynamics based on population pyramids**

Max Hahn-Klimroth^+^, João Pedro Meireles^+^, Laurie Bingaman Lackey, Nick van Eeuwijk, Mads F. Bertelsen, Paul W. Dierkes, Marcus Clauss

+ These authors contributed equally.

**Supplementary material *and/or* additional information**

***Table S1:*** *Frequency (counts) of shape series.*

| Length (years) | 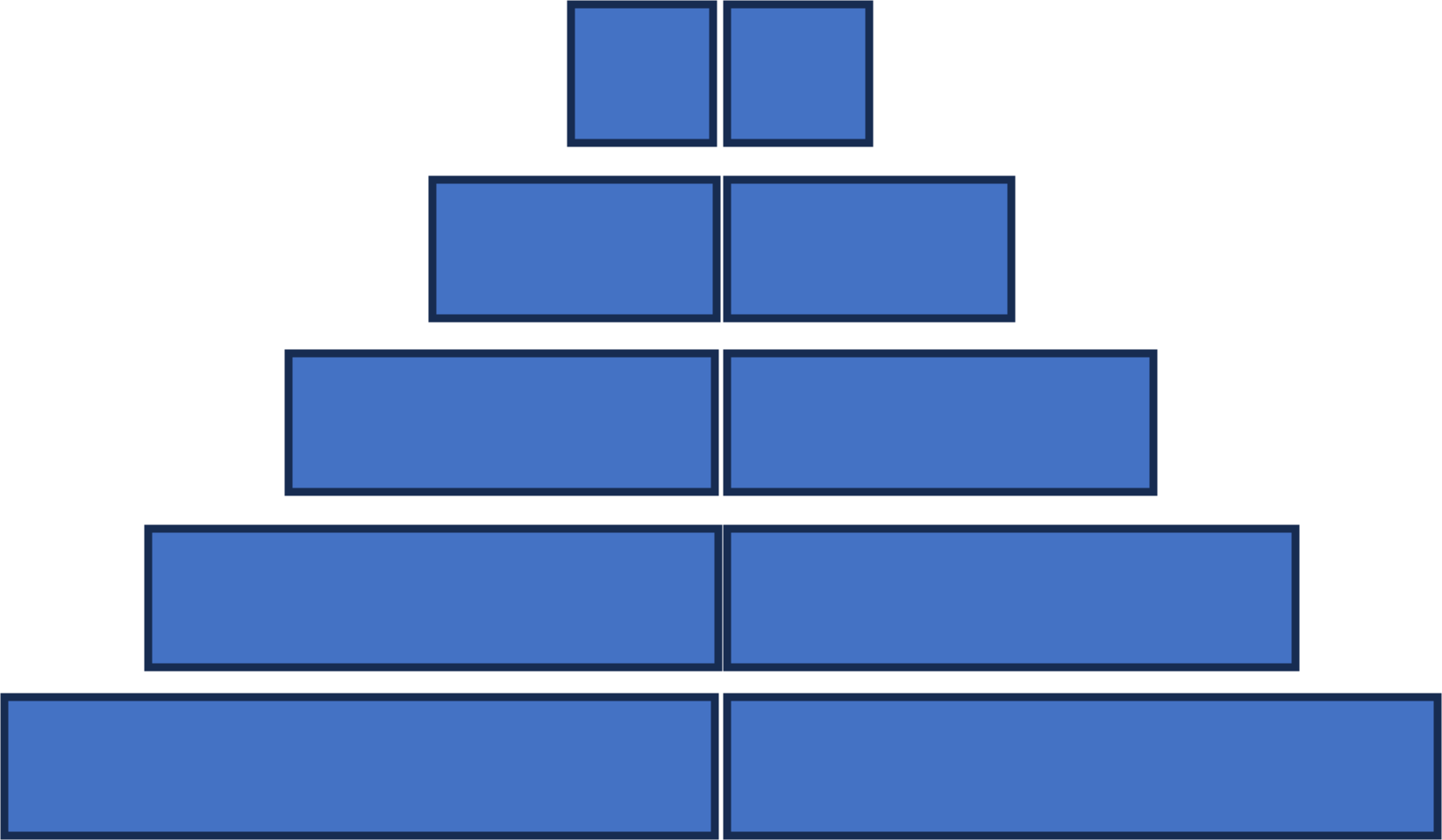 | 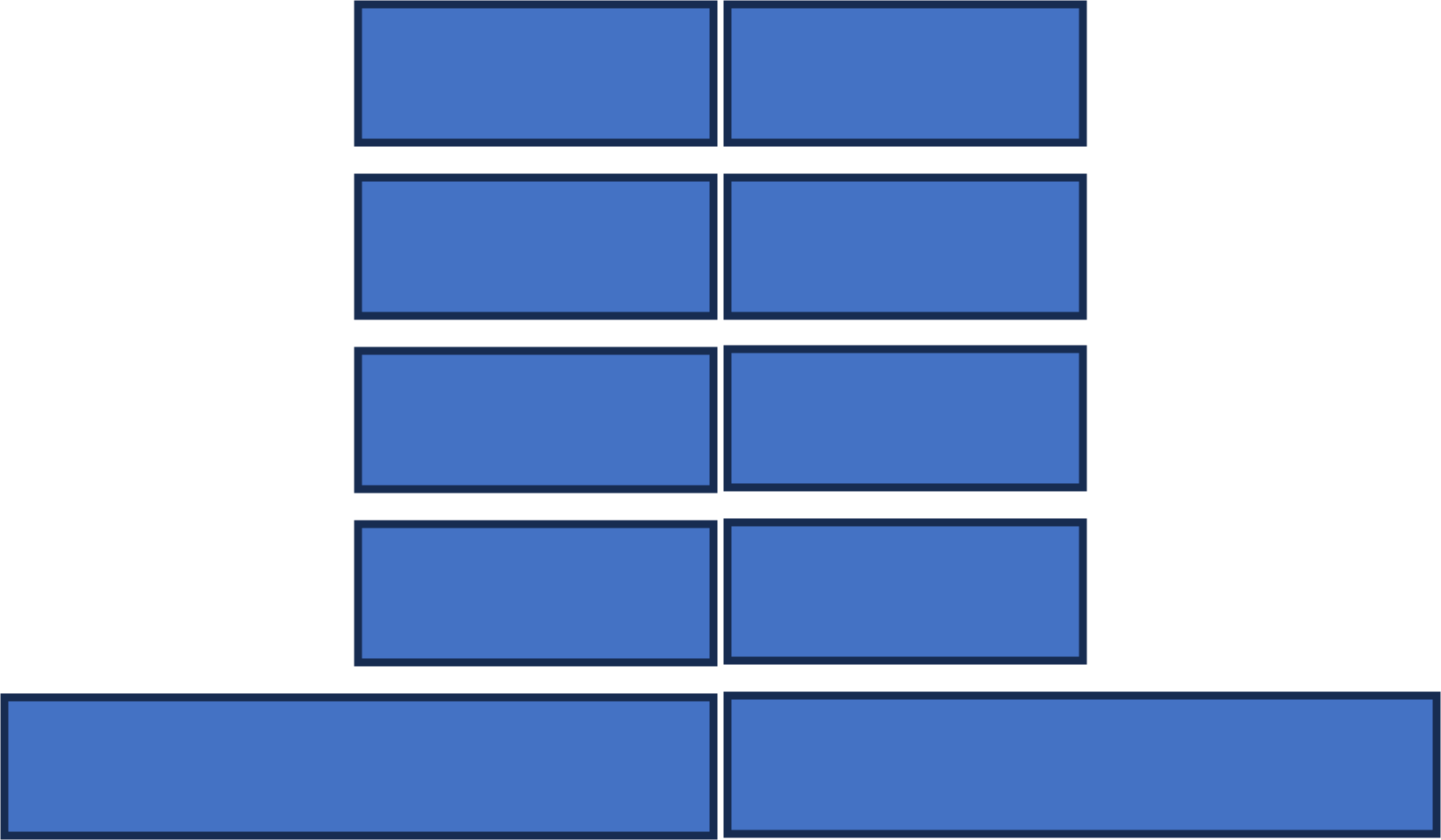 | 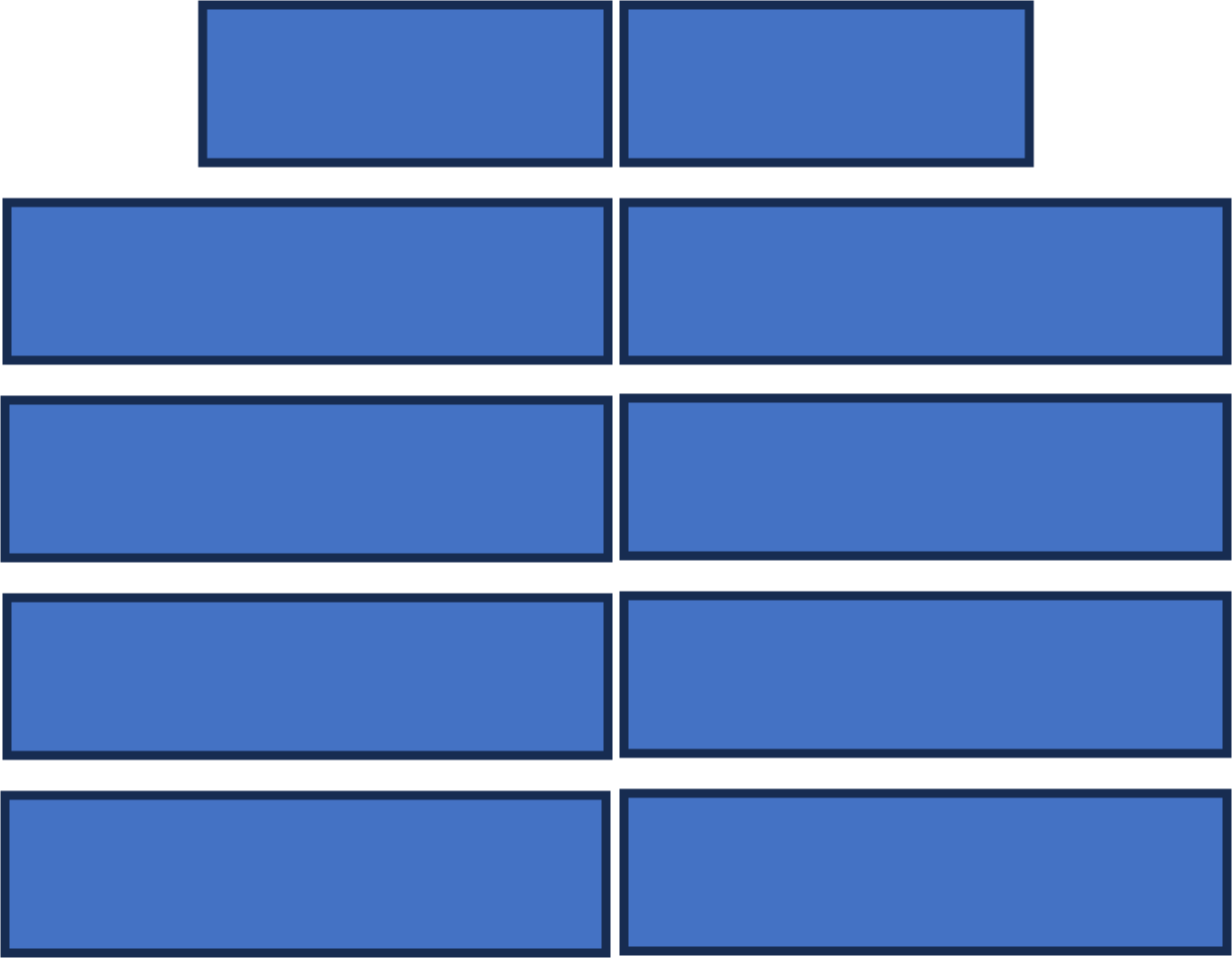 | 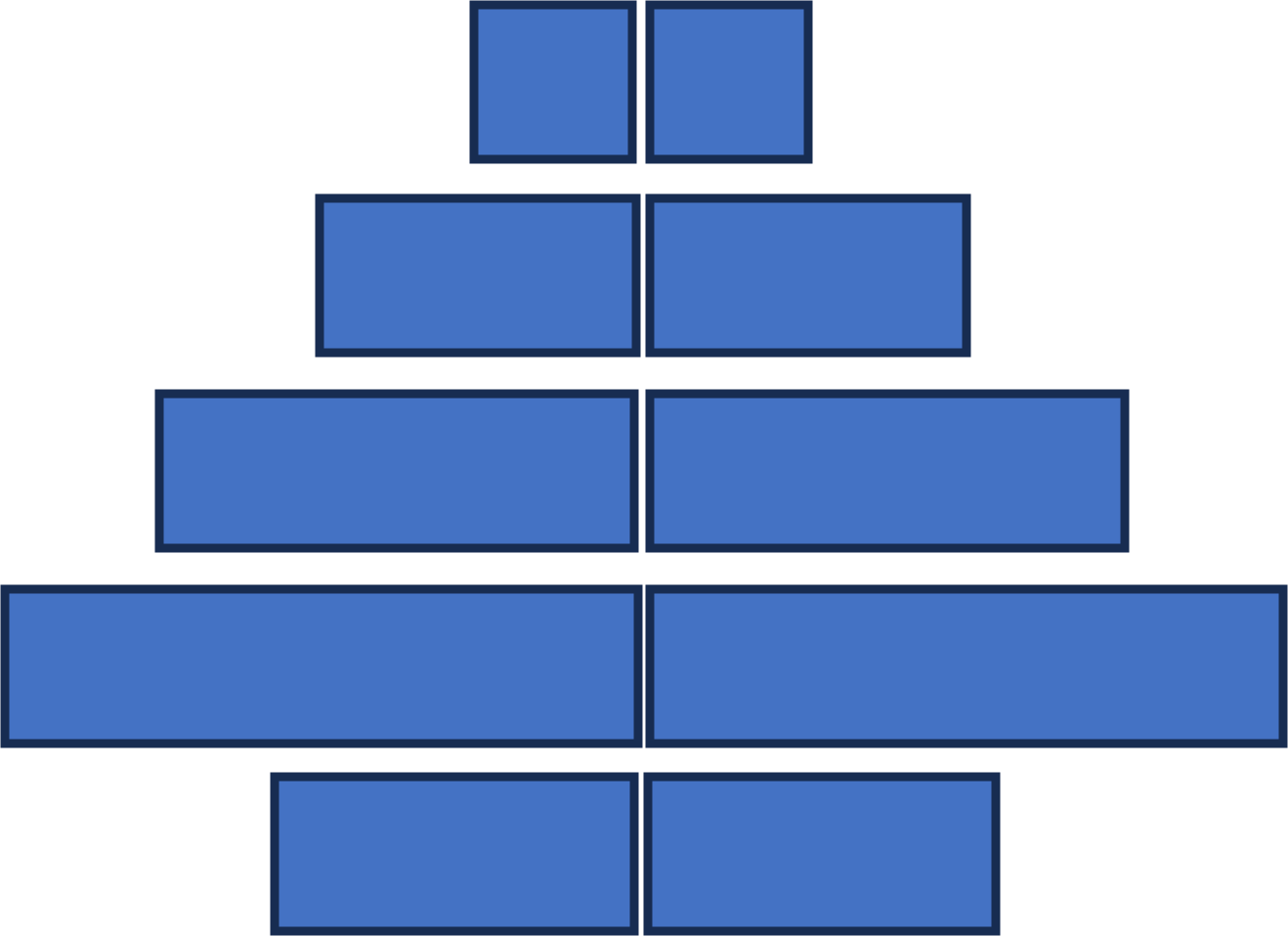 | 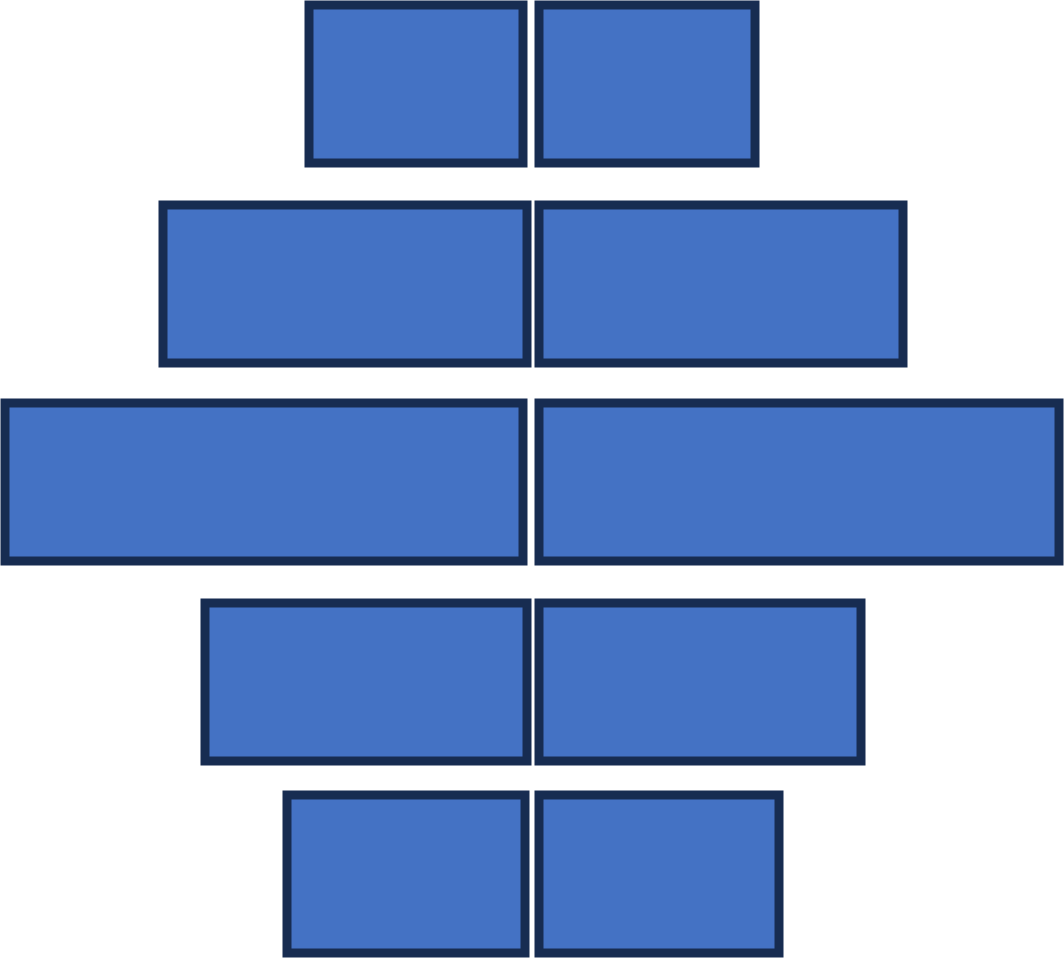 | 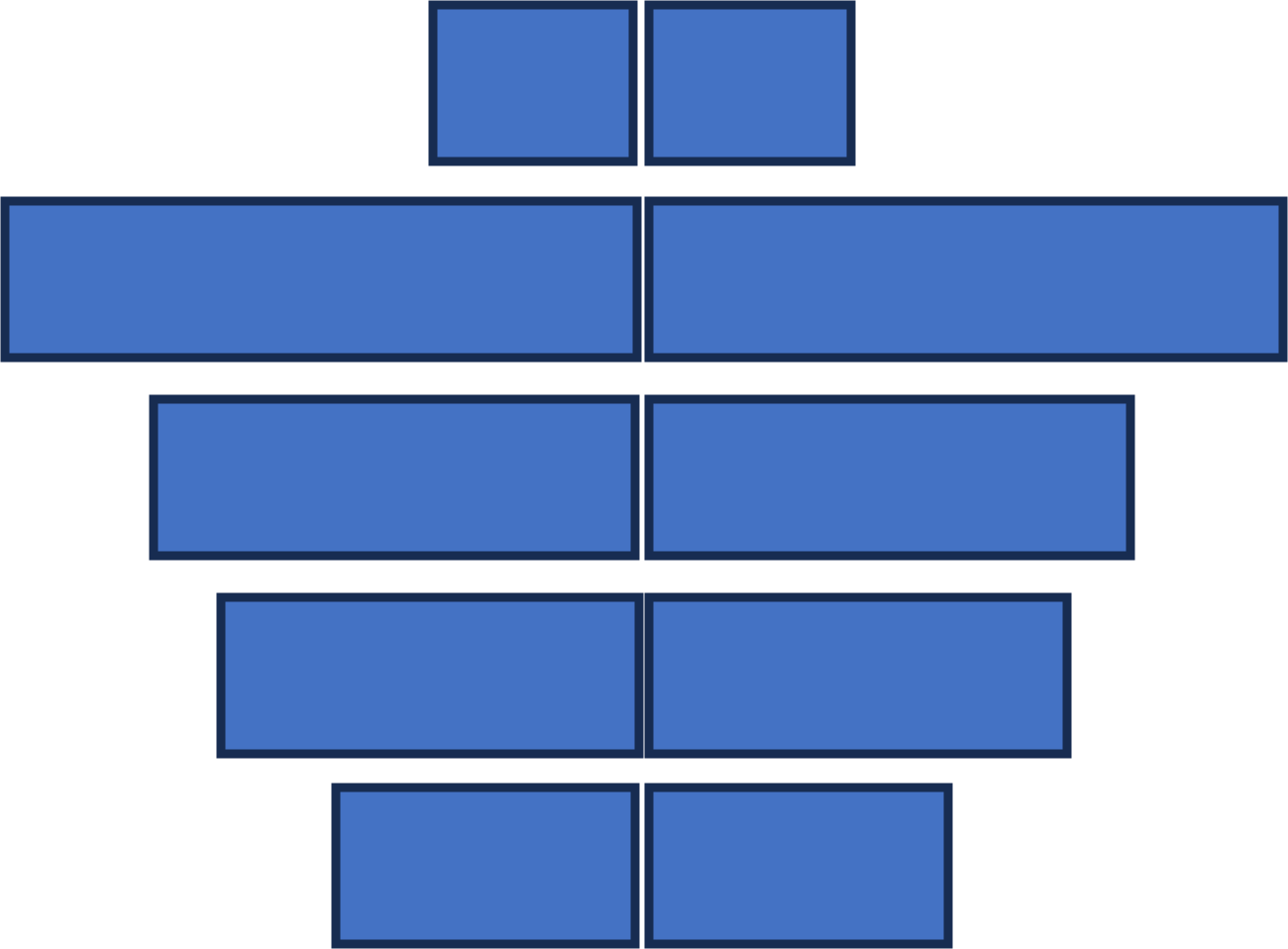 | 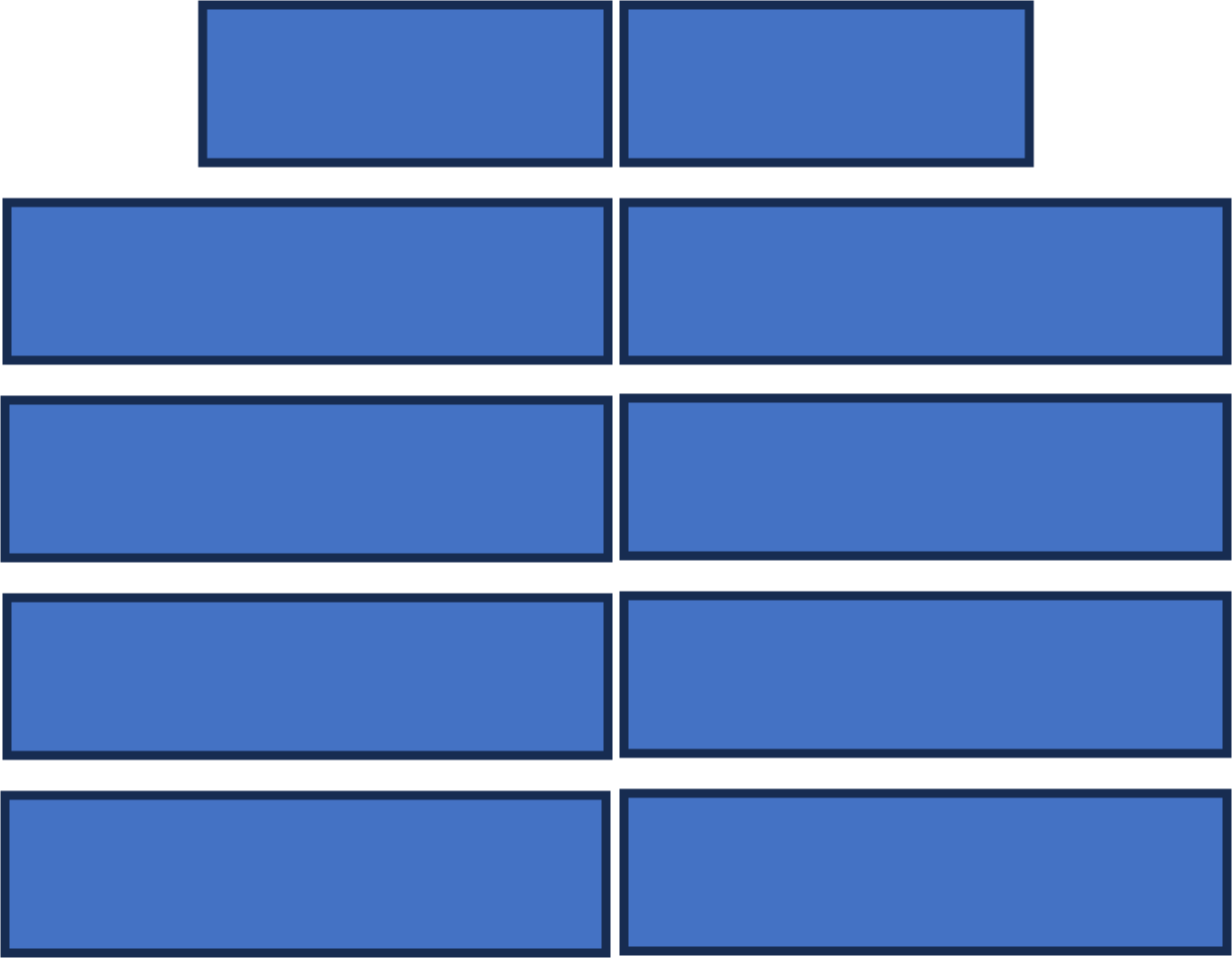 | 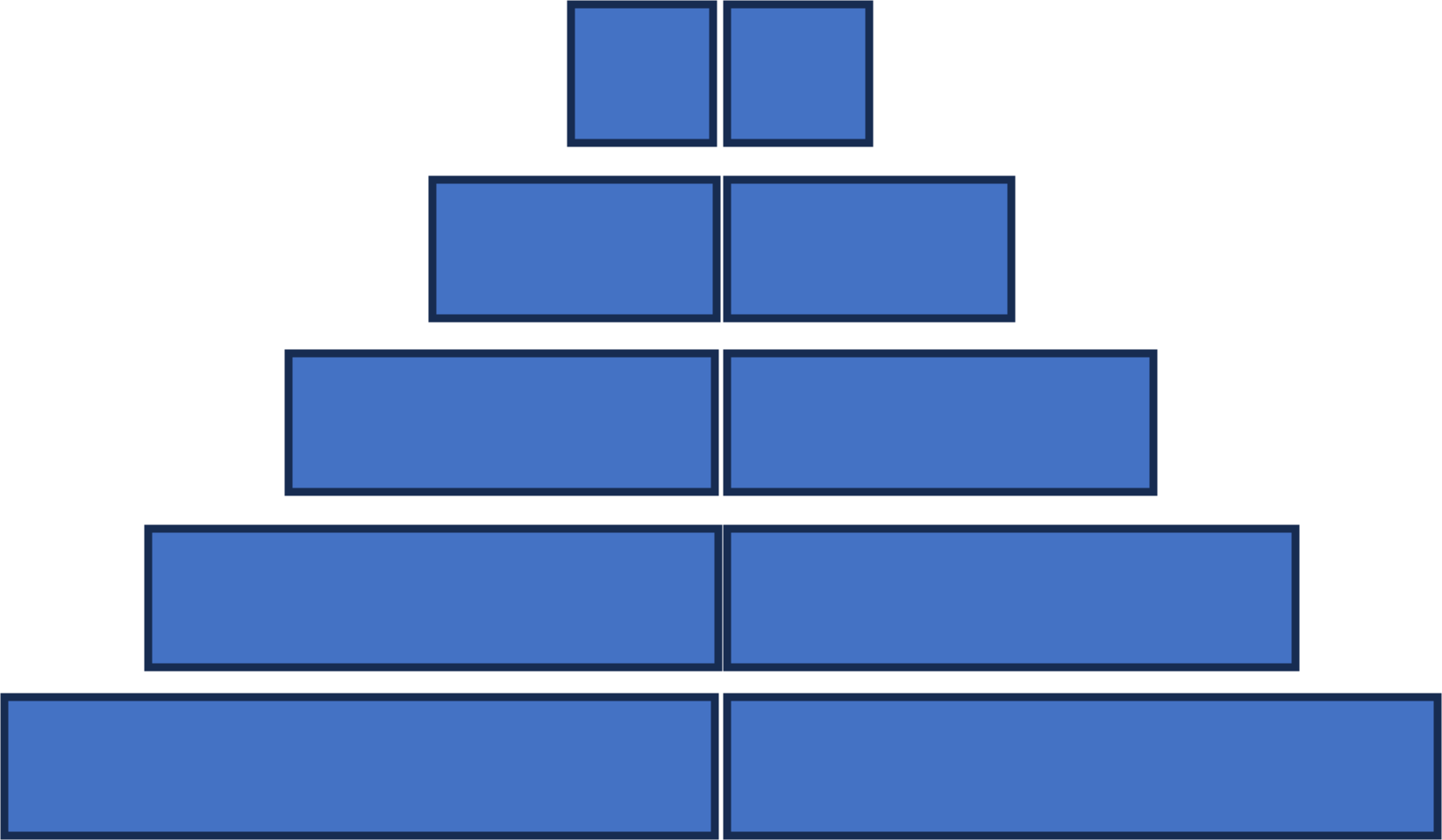 | 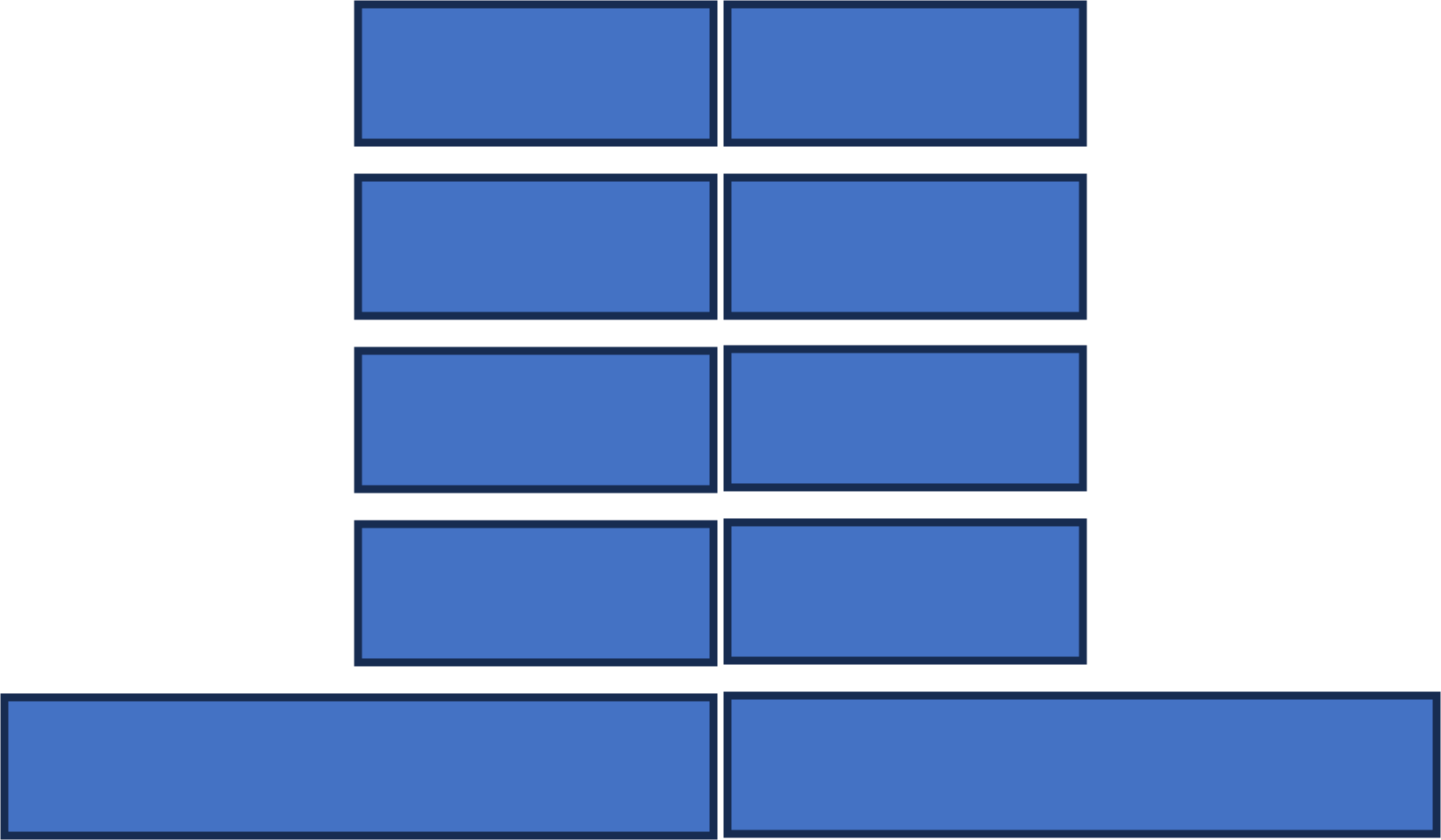 | 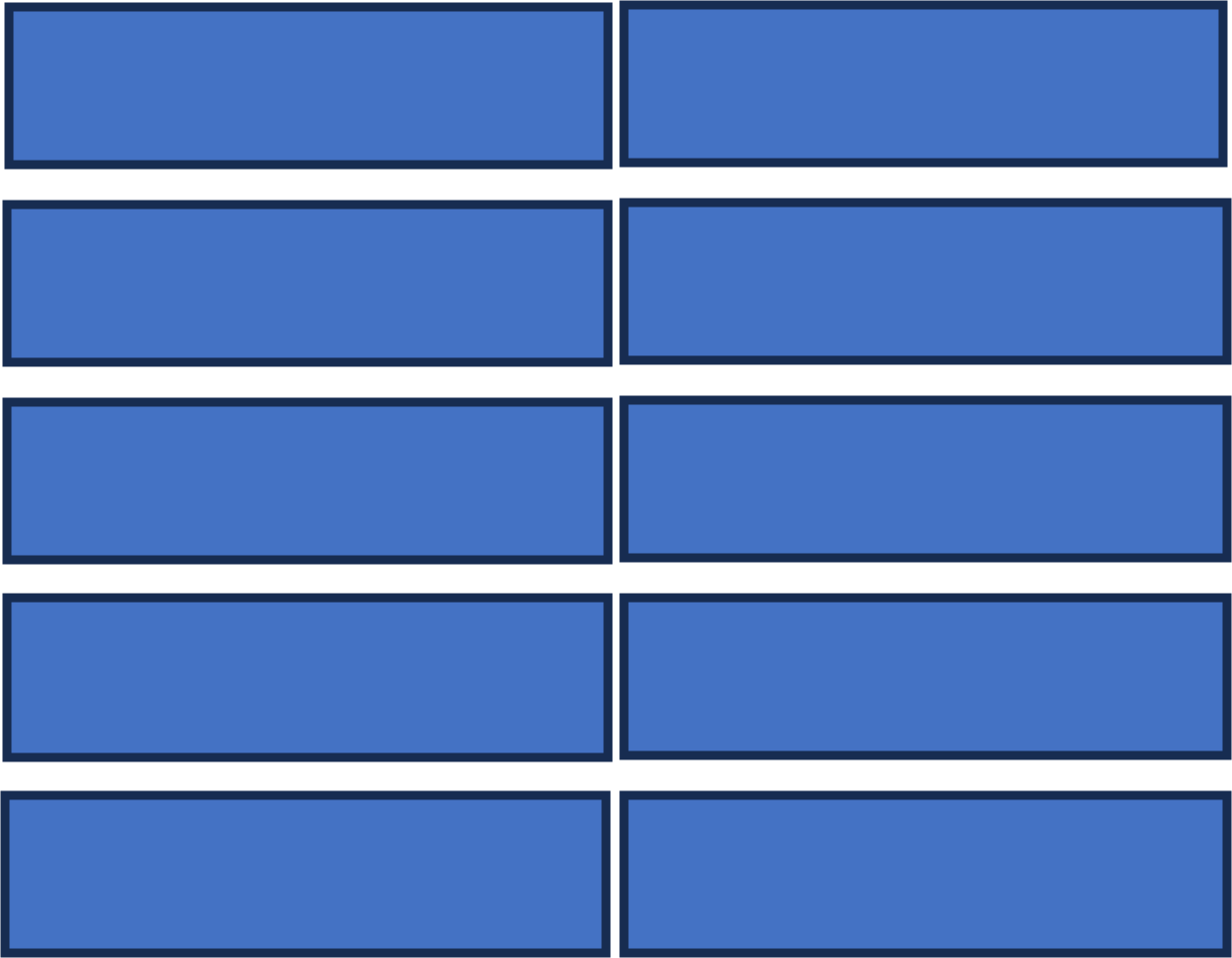 | 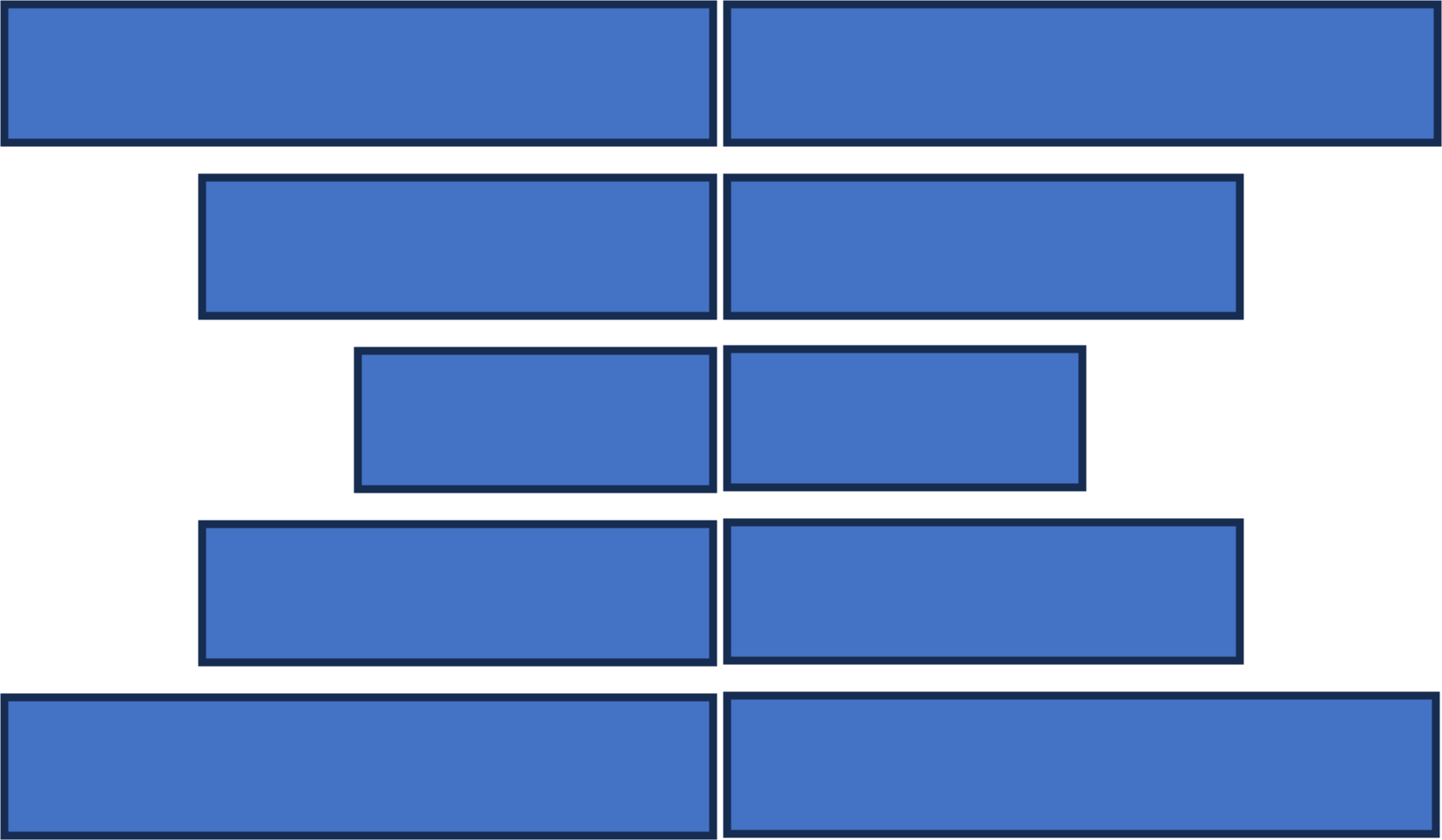 |
| --- | --- | --- | --- | --- | --- | --- | --- | --- | --- | --- | --- |
| **2** | 1184 | 212 | 775 | 1040 | 521 | 264 | 19 | 6 | 20 | 18 | 149 |
| **3** | 780 | 51 | 237 | 328 | 167 | 136 | 7 | 11 | 4 | 2 | 40 |
| **4** | 551 | 18 | 85 | 123 | 54 | 59 | 1 | - | 2 | 1 | 18 |
| **5** | 365 | 6 | 30 | 34 | 10 | 32 | - | - | 1 | - | 7 |
| **6** | 276 | 5 | 6 | 8 | 9 | 22 | - | - | 1 | - | 1 |
| **7** | 192 | 1 | 2 | 6 | 2 | 9 | - | - | - | - | 2 |
| **8** | 144 | - | 1 | - | - | 8 | - | 1 | - | - | - |
| **9** | 125 | 1 | - | - | - | 5 | - | - | - | - | - |
| **10** | 80 | - | - | - | - | 3 | - | - | - | - | 1 |
| **11** | 91 | 1 | - | - | - | 2 | - | - | - | - | 1 |
| **12** | 71 | - | - | - | - | - | - | - | - | - | - |
| **13** | 62 | - | - | - | - | 2 | - | - | - | - | - |
| **14** | 47 | - | - | - | - | 1 | - | - | - | - | - |
| **15** | 39 | - | - | - | - | - | - | - | - | - | - |
| **16** | 37 | - | - | - | - | - | - | - | - | - | - |
| **17** | 29 | - | - | - | - | - | - | - | - | - | - |
| **18** | 25 | - | - | - | - | - | - | - | - | - | - |
| **19** | 22 | - | - | - | - | - | - | - | - | - | - |
| **20** | 22 | - | - | - | - | - | - | - | - | - | - |

***Table S2:*** Average (±SD if n ≥ 2) population change from start to end of shape series.

| Length (years) | 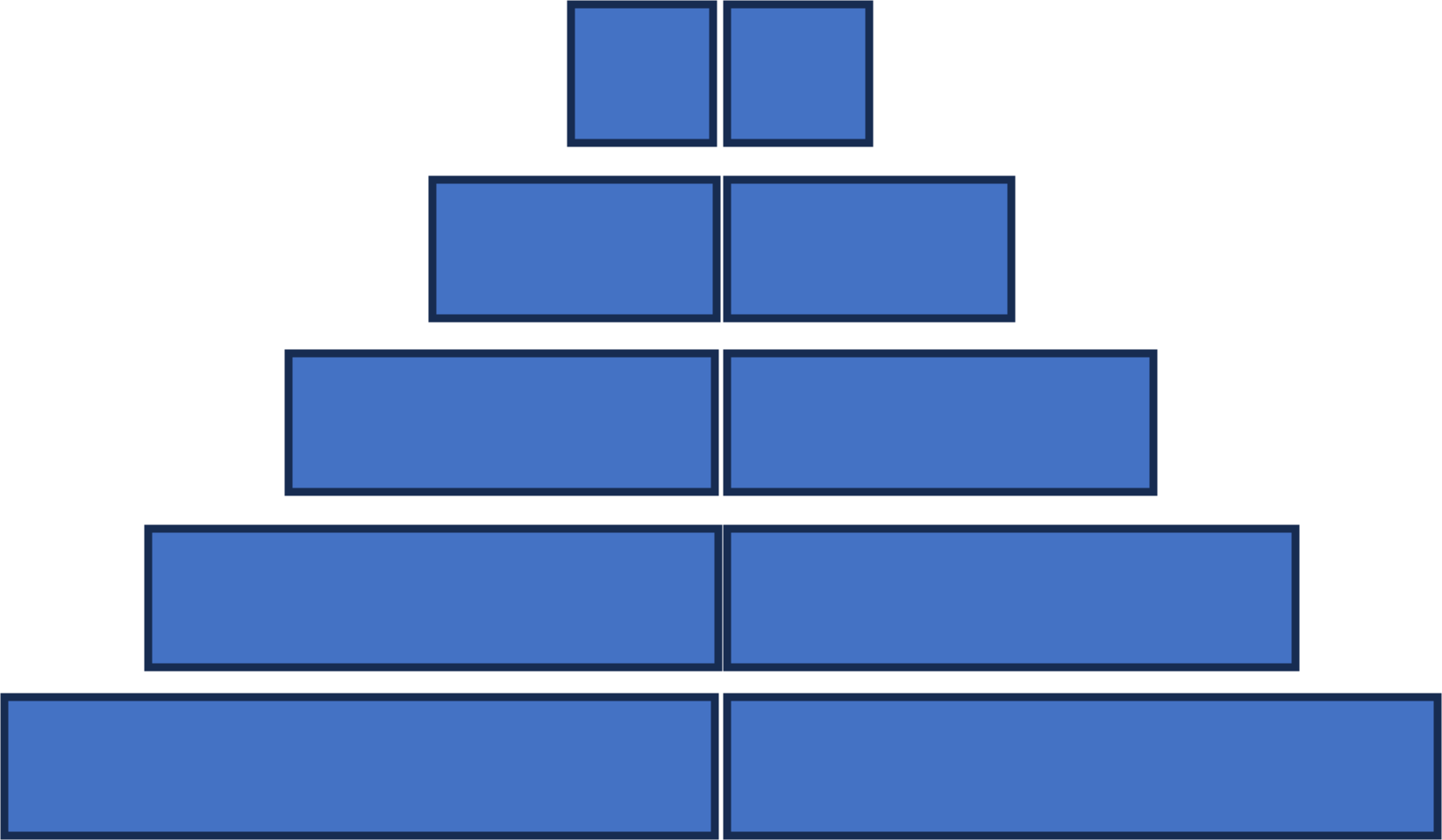 | 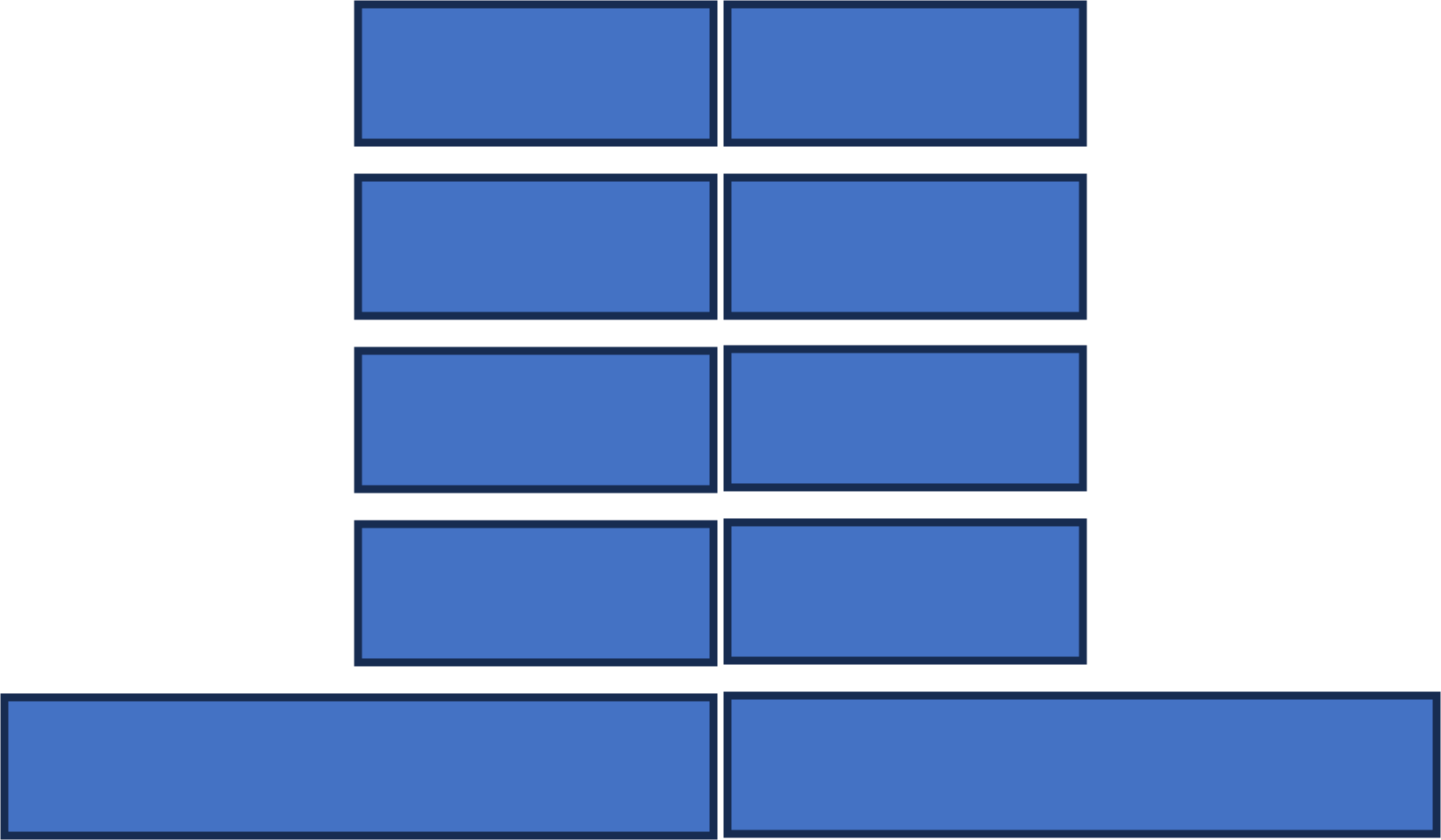 | 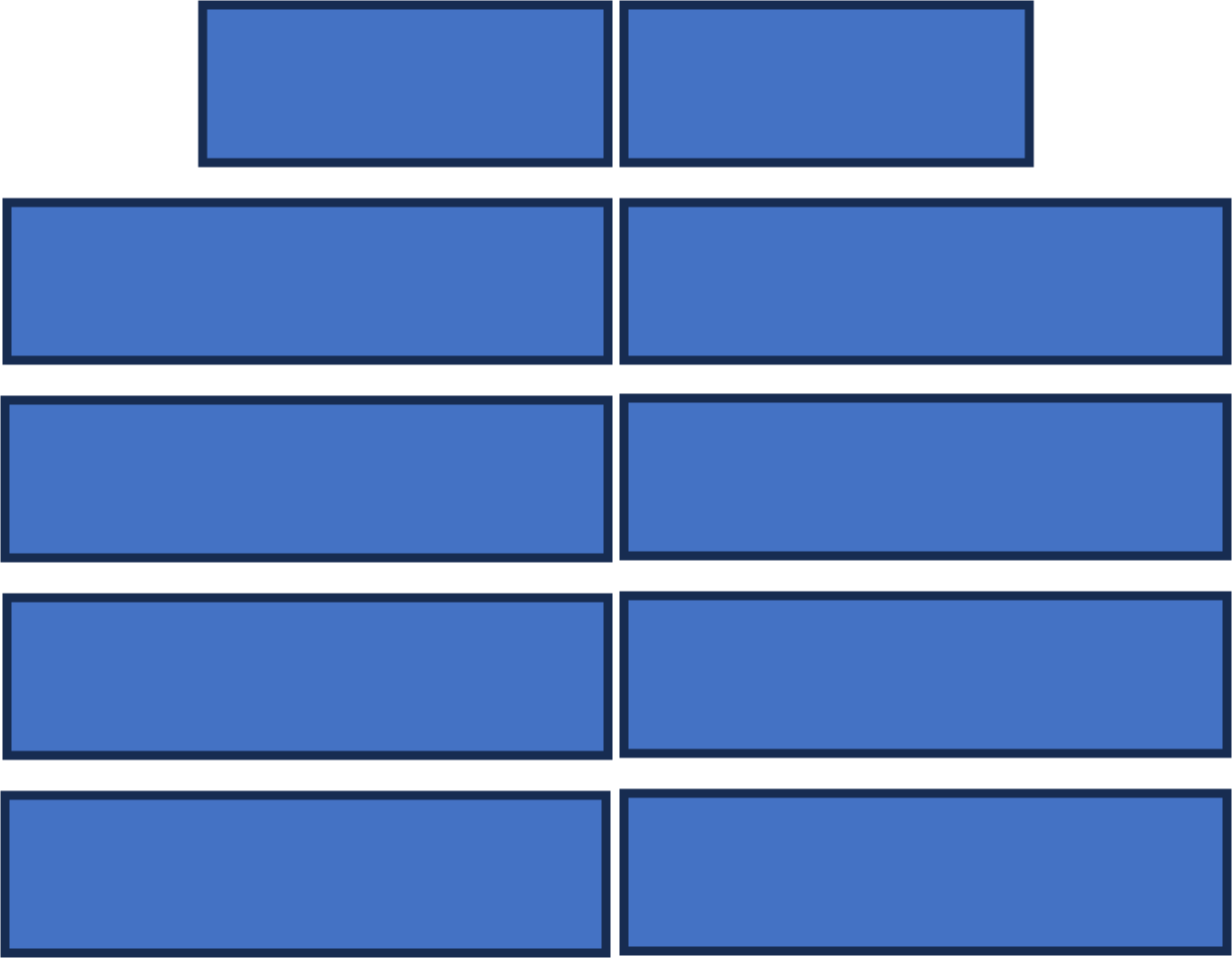 | 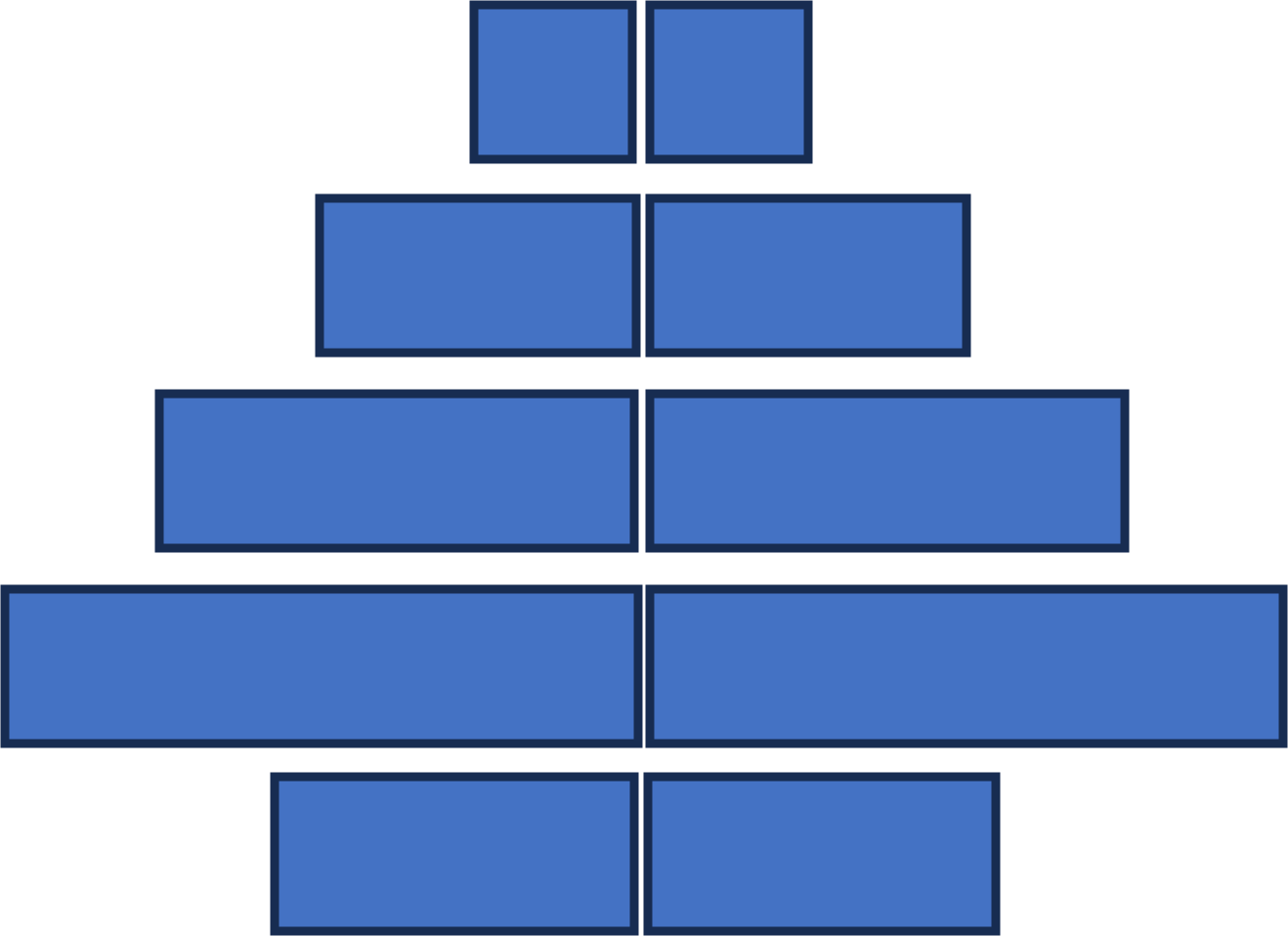 | 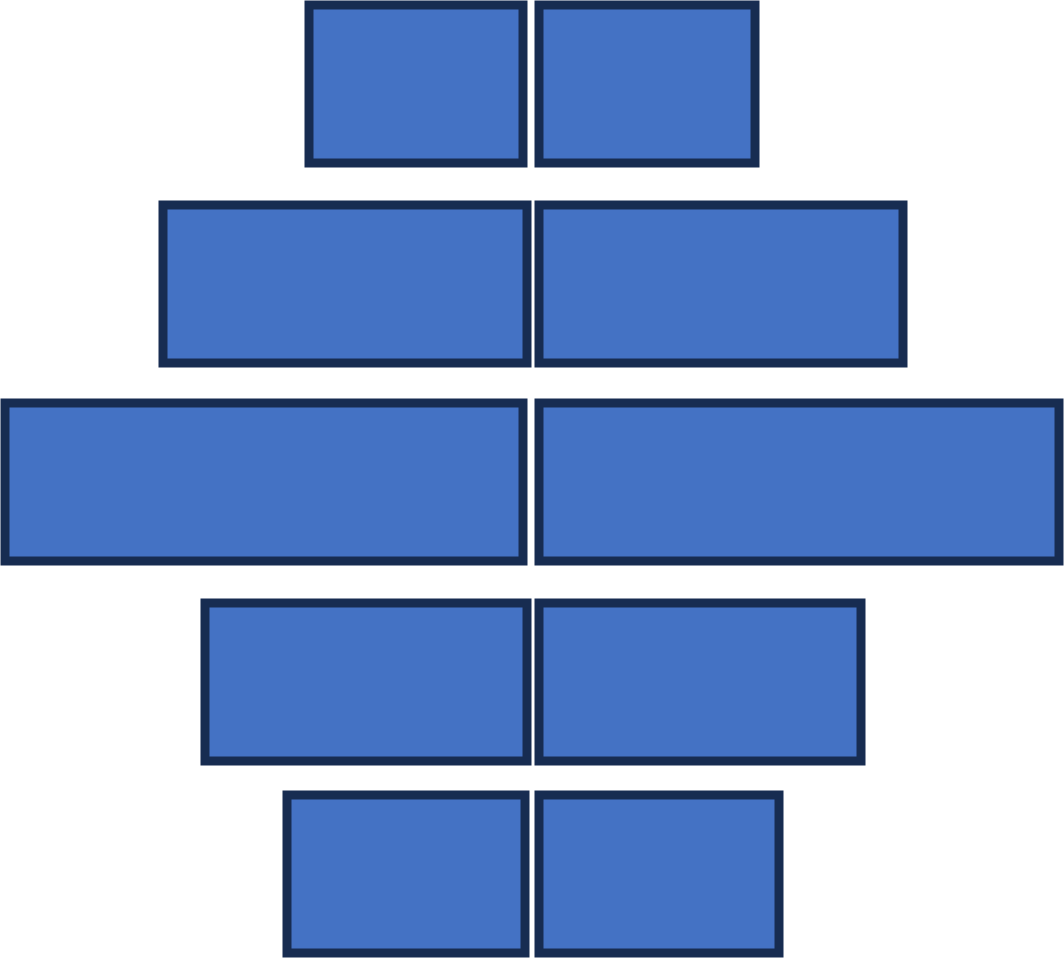 | 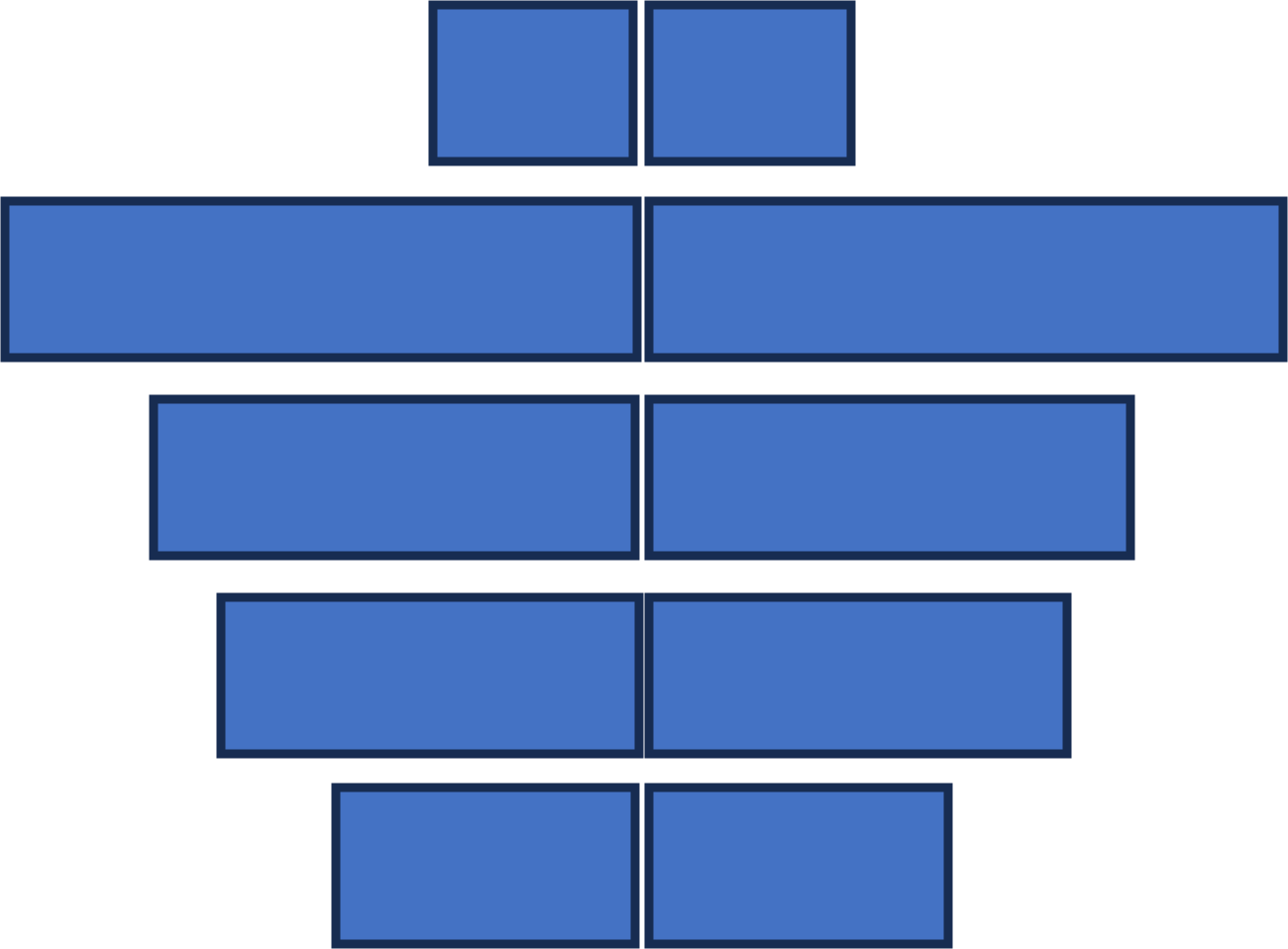 | 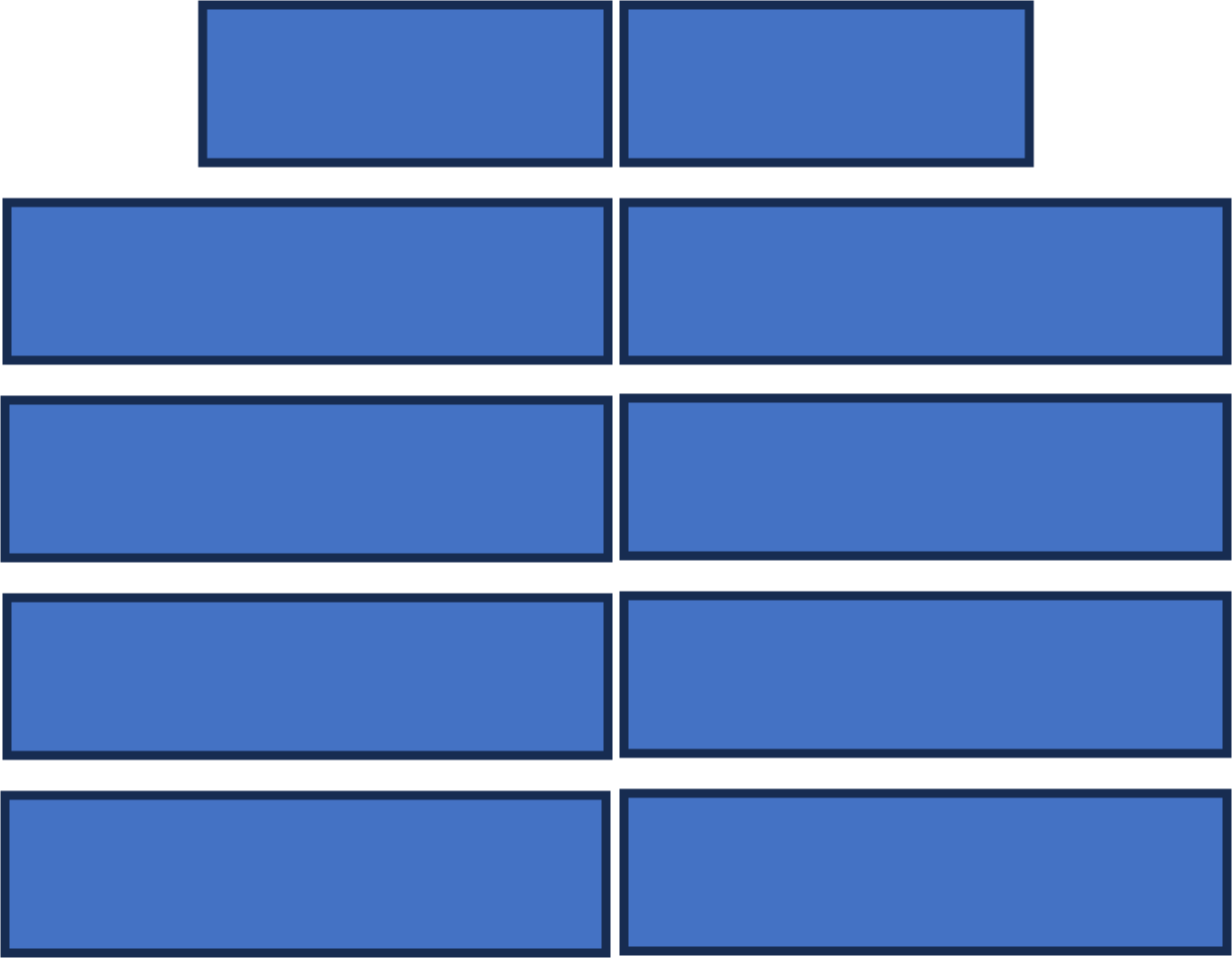 | 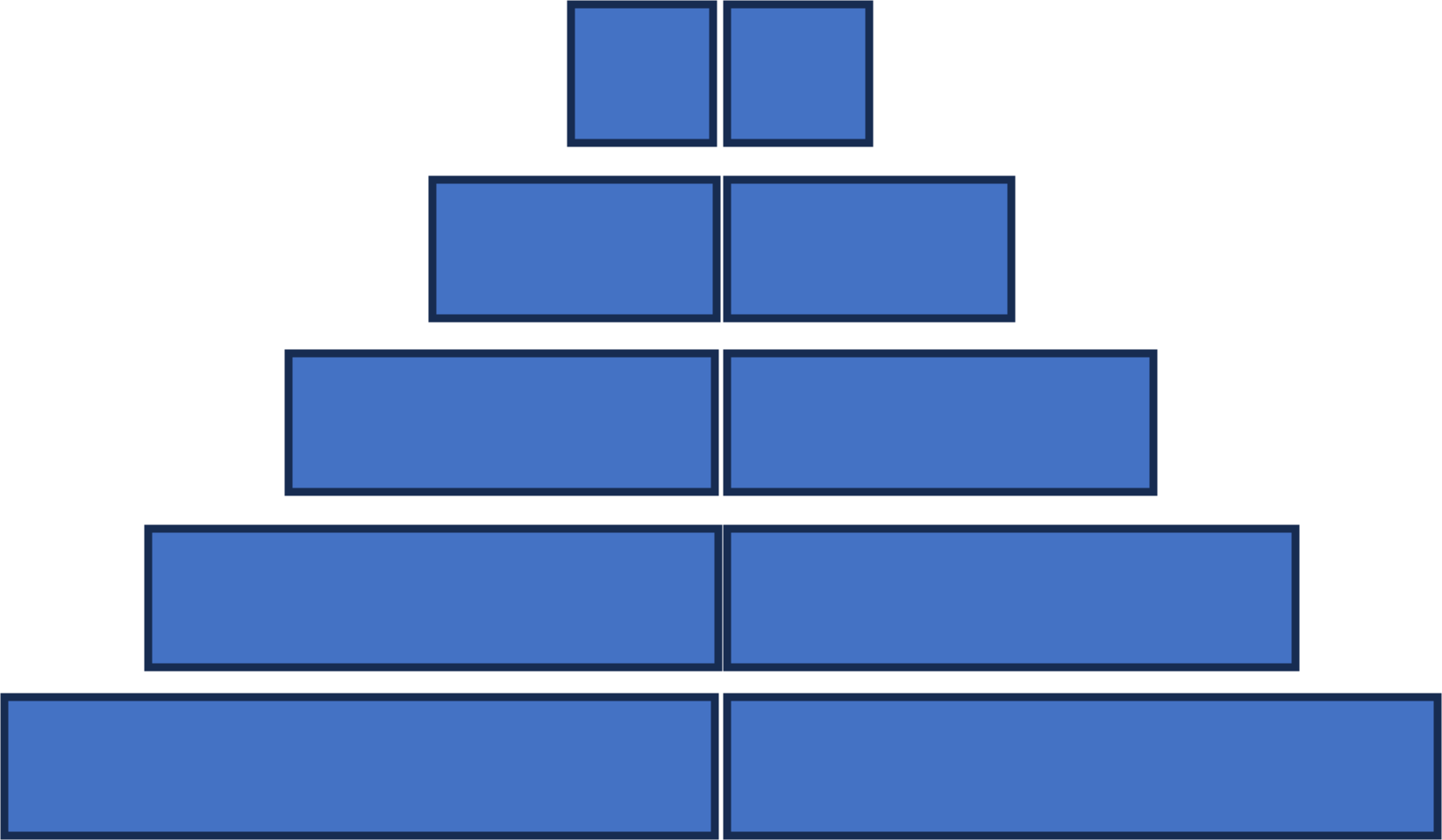 | 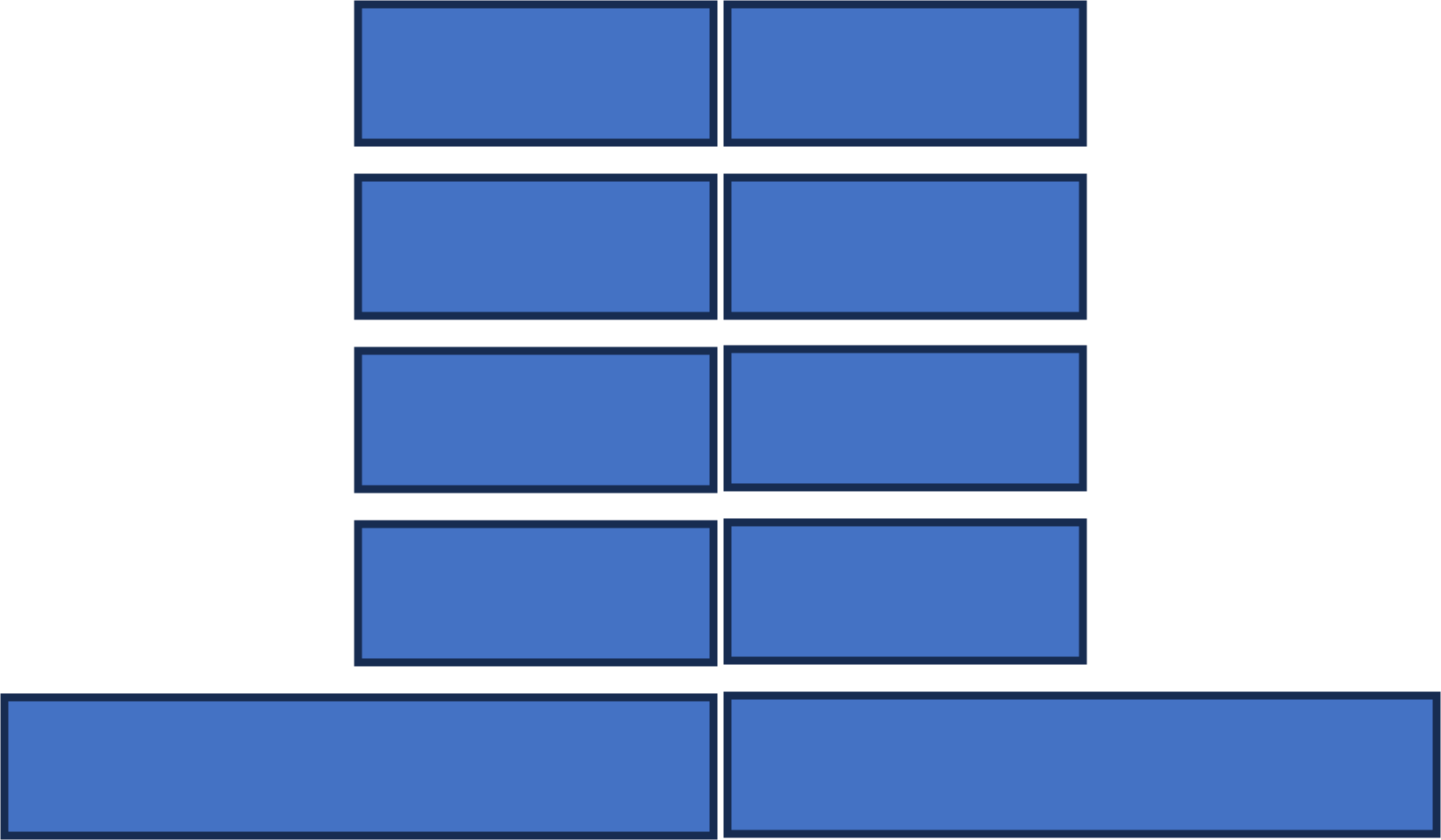 | 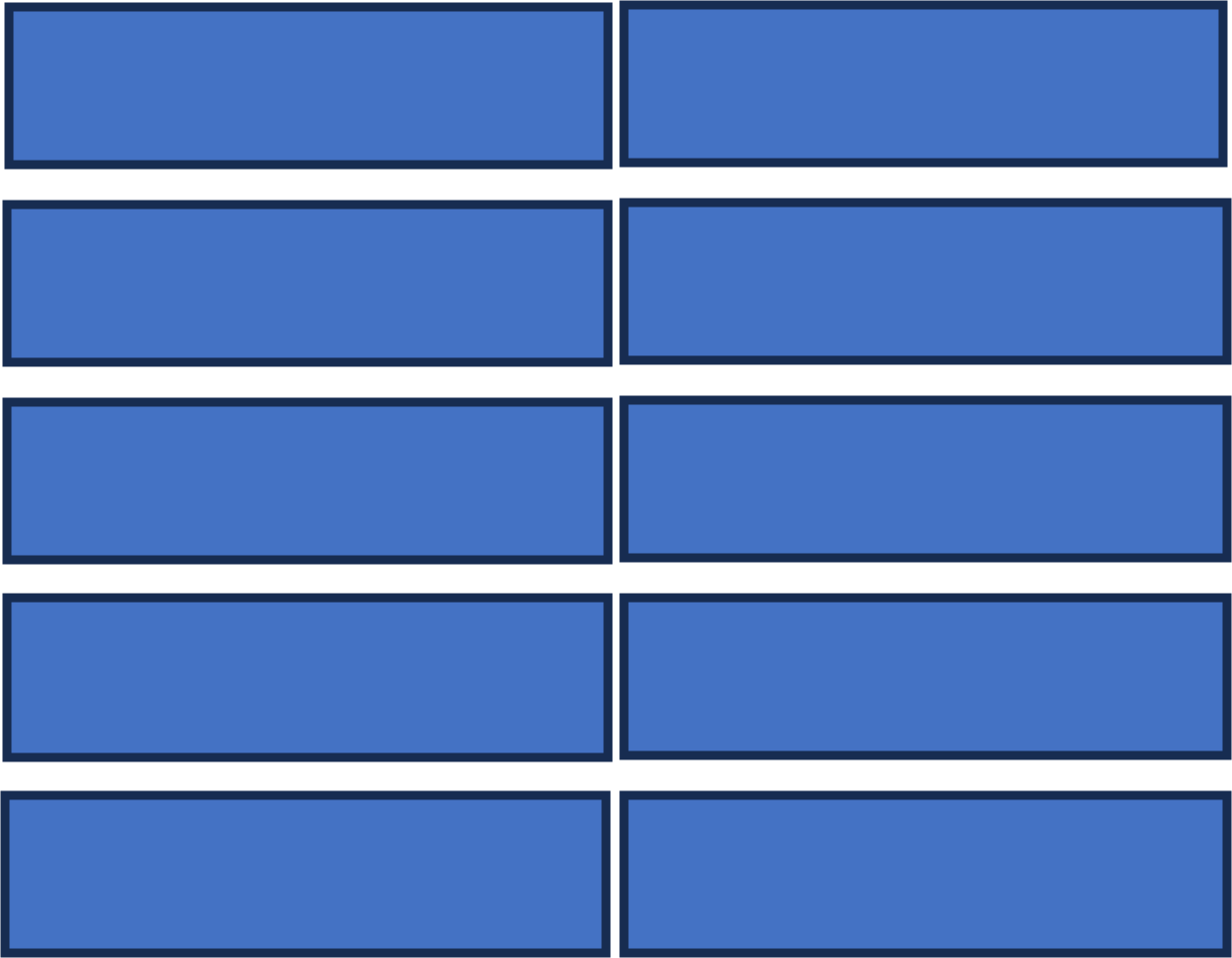 | 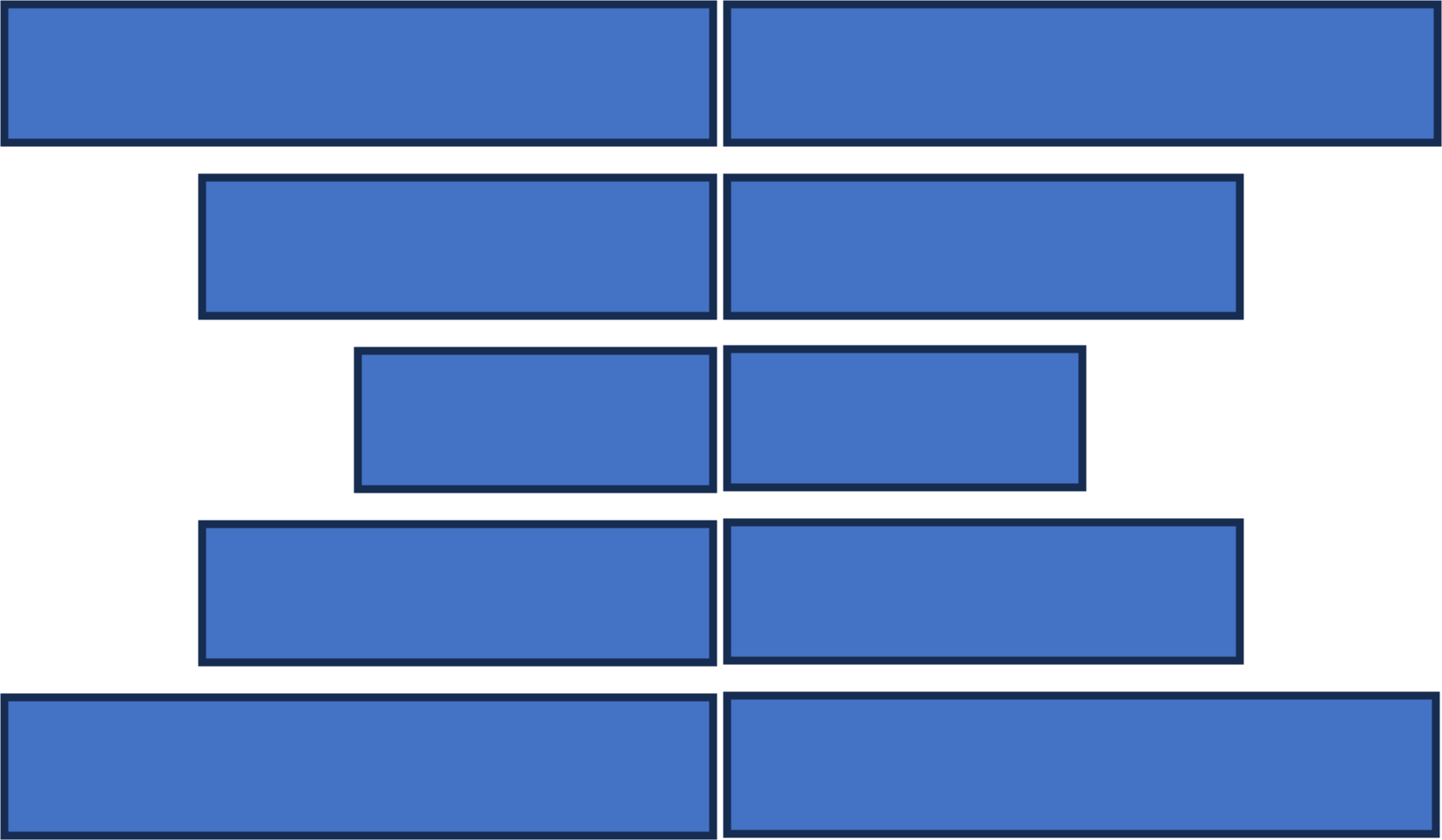 |
| --- | --- | --- | --- | --- | --- | --- | --- | --- | --- | --- | --- |
| **2** | 4.4% (±14.5) | 5.3% (±16.3) | -0.1% (±9.4) | -3.7% (±8.1) | -3.8% (±8.1) | -4.2% (±9.0) | -10.5% (±6.9) | -8.1% (±6.5) | -3.1% (±6.4) | -1.8% (±6.2) | 4.0% (±19.7) |
| **3** | 12.3% (±23.0) | 13.9% (±26.4) | 0.2% (±12.5) | -5.6% (±10.8) | -7.6% (±11.3) | -8.2% (±11.9) | -14.5% (±9.1) | -17.2% (±11.4) | -11.3% (±9.2) | -3.9% (±3.9) | 8.8% (±21.7) |
| **4** | 24.0% (±46.4) | 24.0% (±63.6) | -1.0% (±15.4) | -6.7% (±12.4) | -10.6% (±13.3) | -13.8% (±12.5) | -5.6% | - | -11.2% (±7.4) | -31.2% | 7.0% (±37.9) |
| **5** | 28.3% (±43.0) | 67.0% (±95.4) | 5.2% (±25.9) | -6.9% (±11.3) | -8.0% (±18.8) | -17.8% (±13.7) | - | - | -38.9% | - | 30.4% (±49.9) |
| **6** | 42.5% (±53.1) | 27.5% (±41.5) | -6.2% (±18.4) | 3.3% (±8.6) | -10.1% (±12.0) | -11.3% (±17.5) | - | - | 50.0% | - | -27.4% |
| **7** | 49.2% (±64.1) | 23.9% | 36.4% (±40.0) | 7.3% (±13.4) | -2.9% (±10.5) | -20.4% (±14.7) | - | - | - | - | -44.7% (±7.5) |
| **8** | 49.0% (±70.6) | - | 36.4% | - | - | -24.8% (±14.4) | - | -64.0% | - | - | - |
| **9** | 74.8% (±87.9) | 84.6% | - | - | - | -40.0% (±20.2) | - | - | - | - | - |
| **10** | 78.1% (±86.9) | - | - | - | - | -15.2% (±12.4) | - | - | - | - | -48.3% |
| **11** | 91.2% (±103.4) | 190.0% | - | - | - | -28.2% (±14.0) | - | - | - | - | -14.6% |
| **12** | 123.5% (±133.9) | - | - | - | - | - | - | - | - | - | - |
| **13** | 119.0% (±144.0) | - | - | - | - | -17.7% (±2.9) | - | - | - | - | - |
| **14** | 125.3% (±104.6) | - | - | - | - | -41.0% | - | - | - | - | - |
| **15** | 127.8% (±135.8) | - | - | - | - | - | - | - | - | - | - |
| **16** | 140.9% (±164.5) | - | - | - | - | - | - | - | - | - | - |
| **17** | 169.1% (±176.8) | - | - | - | - | - | - | - | - | - | - |
| **18** | 295.3% (±311.2) | - | - | - | - | - | - | - | - | - | - |
| **19** | 134.4% (±136.8) | - | - | - | - | - | - | - | - | - | - |
| **20** | 311.9% (±393.3) | - | - | - | - | - | - | - | - | - | - |

***Table S3:*** *Number of shape transitions (total n=54081).*

| From/to | **pyramid** | **plunger** | **bell** | **lower diamond** | **middle diamond** | **upper diamond** | **inverted bell** | **inverted pyramid** | **inverted plunger** | **column** | **hourglass** |
| --- | --- | --- | --- | --- | --- | --- | --- | --- | --- | --- | --- |
| **pyramid** | 27198 | 521 | 1860 | 3066 | 420 | 114 | 3 | 1 | 9 | 11 | 243 |
| **plunger** | 631 | 441 | 70 | 206 | 13 | 5 | 4 | 0 | 3 | 17 | 105 |
| **bell** | 2075 | 122 | 1673 | 625 | 597 | 314 | 18 | 6 | 6 | 36 | 126 |
| **lower diamond** | 1719 | 52 | 982 | 2277 | 1078 | 167 | 23 | 3 | 1 | 13 | 45 |
| **middle diamond** | 495 | 23 | 646 | 212 | 1114 | 654 | 26 | 4 | 3 | 12 | 41 |
| **upper diamond** | 231 | 44 | 347 | 59 | 187 | 1185 | 38 | 25 | 24 | 18 | 155 |
| **inverted bell** | 5 | 6 | 10 | 18 | 21 | 15 | 36 | 9 | 7 | 14 | 13 |
| **inverted pyramid** | 0 | 1 | 1 | 2 | 3 | 4 | 12 | 35 | 13 | 3 | 12 |
| **inverted plunger** | 12 | 7 | 7 | 6 | 2 | 5 | 5 | 8 | 43 | 4 | 21 |
| **column** | 16 | 17 | 32 | 19 | 10 | 6 | 9 | 3 | 7 | 25 | 12 |
| **hourglass** | 265 | 125 | 69 | 153 | 28 | 62 | 19 | 10 | 23 | 22 | 347 |

***Table S4:*** *Percentage of shape transitions (total n=54081).*

| From/to | **pyramid** | **plunger** | **bell** | **lower diamond** | **middle diamond** | **upper diamond** | **inverted bell** | **inverted pyramid** | **inverted plunger** | **column** | **hourglass** |
| --- | --- | --- | --- | --- | --- | --- | --- | --- | --- | --- | --- |
| **pyramid** | 50.3% | 1.0% | 3.4% | 5.7% | 0.8% | 0.2% | 0.0% | 0.0% | 0.0% | 0.0% | 0.4% |
| **plunger** | 1.2% | 0.8% | 0.1% | 0.4% | 0.0% | 0.0% | 0.0% | 0.0% | 0.0% | 0.0% | 0.2% |
| **bell** | 3.8% | 0.2% | 3.1% | 1.2% | 1.1% | 0.6% | 0.0% | 0.0% | 0.0% | 0.1% | 0.2% |
| **lower diamond** | 3.2% | 0.1% | 1.8% | 4.2% | 2.0% | 0.3% | 0.0% | 0.0% | 0.0% | 0.0% | 0.1% |
| **middle diamond** | 0.9% | 0.0% | 1.2% | 0.4% | 2.1% | 1.2% | 0.0% | 0.0% | 0.0% | 0.0% | 0.1% |
| **upper diamond** | 0.4% | 0.1% | 0.6% | 0.1% | 0.3% | 2.2% | 0.1% | 0.0% | 0.0% | 0.0% | 0.3% |
| **inverted bell** | 0.0% | 0.0% | 0.0% | 0.0% | 0.0% | 0.0% | 0.1% | 0.0% | 0.0% | 0.0% | 0.0% |
| **inverted pyramid** | 0.0% | 0.0% | 0.0% | 0.0% | 0.0% | 0.0% | 0.0% | 0.1% | 0.0% | 0.0% | 0.0% |
| **inverted plunger** | 0.0% | 0.0% | 0.0% | 0.0% | 0.0% | 0.0% | 0.0% | 0.0% | 0.1% | 0.0% | 0.0% |
| **column** | 0.0% | 0.0% | 0.1% | 0.0% | 0.0% | 0.0% | 0.0% | 0.0% | 0.0% | 0.0% | 0.0% |
| **hourglass** | 0.5% | 0.2% | 0.1% | 0.3% | 0.1% | 0.1% | 0.0% | 0.0% | 0.0% | 0.0% | 0.6% |

***Table S5*** *Average population change per shape transition.*

| From/to | **pyramid** | **plunger** | **bell** | **lower diamond** | **middle diamond** | **upper diamond** | **inverted bell** | **inverted pyramid** | **inverted plunger** | **column** | **hourglass** |
| --- | --- | --- | --- | --- | --- | --- | --- | --- | --- | --- | --- |
| **pyramid** | 7.2% | 5.3% | -2.9% | -4.0% | -6.8% | -6.0% | -12.0% | -7.7% | -3.5% | -6.3% | 2.5% |
| **plunger** | 8.8% | 6.2% | -4.4% | -4.1% | -5.4% | -12.2% | -8.1% | - | -9.4% | -5.3% | 3.7% |
| **bell** | 7.1% | 3.0% | 0.1% | -4.1% | -4.0% | -5.2% | -5.4% | -7.0% | -14.5% | -2.0% | -0.1% |
| **lower diamond** | 9.5% | 7.6% | 2.2% | -3.4% | -3.9% | -5.7% | -5.8% | -9.1% | -9.1% | -4.1% | 3.6% |
| **middle diamond** | 7.2% | 4.5% | 2.1% | -4.3% | -3.9% | -4.9% | -8.1% | -14.8% | -9.3% | -2.4% | -0.6% |
| **upper diamond** | 4.4% | 2.3% | 0.9% | -7.6% | -5.2% | -4.3% | -6.7% | -9.2% | -9.1% | -8.2% | -0.9% |
| **inverted bell** | 14.2% | 7.1% | -1.8% | -6.5% | -10.1% | -8.8% | -9.1% | -3.8% | -1.8% | -0.6% | -1.1% |
| **inverted pyramid** | - | -17.6% | 5.6% | -1.5% | -12.3% | -8.5% | -12.8% | -8.9% | -7.9% | -11.7% | -5.4% |
| **inverted plunger** | 19.4% | 1.0% | 2.3% | -9.0% | -8.1% | 9.6% | -10.6% | -10.3% | -3.4% | -3.1% | -2.8% |
| **column** | 2.4% | 4.7% | -1.2% | -6.6% | -5.6% | -5.6% | -4.3% | -10.9% | -1.9% | -2.2% | 0.2% |
| **hourglass** | 8.0% | 4.1% | -0.9% | -5.6% | -10.8% | -6.4% | -8.5% | -11.4% | -5.1% | -4.1% | 4.0% |

***Table S6*** *Frequency (counts) of shape series.*

| Series  of n | **pyramid** | **plunger** | **bell** | **lower diamond** | **middle diamond** | **upper diamond** | **inverted bell** | **inverted pyramid** | **inverted plunger** | **column** | **hourglass** |
| --- | --- | --- | --- | --- | --- | --- | --- | --- | --- | --- | --- |
| 2 | 1184 | 212 | 775 | 1040 | 521 | 264 | 19 | 6 | 20 | 18 | 149 |
| 3 | 780 | 51 | 237 | 328 | 167 | 136 | 7 | 11 | 4 | 2 | 40 |
| 4 | 551 | 18 | 85 | 123 | 54 | 59 | 1 | - | 2 | 1 | 18 |
| 5 | 365 | 6 | 30 | 34 | 10 | 32 | - | - | 1 | - | 7 |
| 6 | 276 | 5 | 6 | 8 | 9 | 22 | - | - | 1 | - | 1 |
| 7 | 192 | 1 | 2 | 6 | 2 | 9 | - | - | - | - | 2 |
| 8 | 144 | - | 1 | - | - | 8 | - | 1 | - | - | - |
| 9 | 125 | 1 | - | - | - | 5 | - | - | - | - | - |
| 10 | 80 | - | - | - | - | 3 | - | - | - | - | 1 |
| 11 | 91 | 1 | - | - | - | 2 | - | - | - | - | 1 |
| 12 | 71 | - | - | - | - | - | - | - | - | - | - |
| 13 | 62 | - | - | - | - | 2 | - | - | - | - | - |
| 14 | 47 | - | - | - | - | 1 | - | - | - | - | - |
| 15 | 39 | - | - | - | - | - | - | - | - | - | - |
| 16 | 37 | - | - | - | - | - | - | - | - | - | - |
| 17 | 29 | - | - | - | - | - | - | - | - | - | - |
| 18 | 25 | - | - | - | - | - | - | - | - | - | - |
| 19 | 22 | - | - | - | - | - | - | - | - | - | - |
| 20 | 22 | - | - | - | - | - | - | - | - | - | - |
| 21 | 18 | - | - | - | - | - | - | - | - | - | - |
| 22 | 16 | - | - | - | - | - | - | - | - | - | - |
| 23 | 20 | - | - | - | - | - | - | - | - | - | - |
| 24 | 14 | - | - | - | - | - | - | - | - | - | - |
| 25 | 18 | - | - | - | - | - | - | - | - | - | - |
| 26 | 15 | - | - | - | - | - | - | - | - | - | - |
| 27 | 11 | - | - | - | - | - | - | - | - | - | - |
| 28 | 8 | - | - | - | - | - | - | - | - | - | - |
| 29 | 7 | - | - | - | - | - | - | - | - | - | - |
| 30 | 5 | - | - | - | - | - | - | - | - | - | - |
| 31 | 15 | - | - | - | - | - | - | - | - | - | - |
| 32 | 15 | - | - | - | - | - | - | - | - | - | - |
| 33 | 11 | - | - | - | - | - | - | - | - | - | - |
| 34 | 9 | - | - | - | - | - | - | - | - | - | - |
| 35 | 10 | - | - | - | - | - | - | - | - | - | - |
| 36 | 10 | - | - | - | - | - | - | - | - | - | - |
| 37 | 6 | - | - | - | - | - | - | - | - | - | - |
| 38 | 8 | - | - | - | - | - | - | - | - | - | - |
| 39 | 7 | - | - | - | - | - | - | - | - | - | - |
| 40 | 2 | - | - | - | - | - | - | - | - | - | - |
| 41 | 12 | - | - | - | - | - | - | - | - | - | - |
| 42 | 5 | - | - | - | - | - | - | - | - | - | - |
| 43 | 3 | - | - | - | - | - | - | - | - | - | - |
| 44 | 6 | - | - | - | - | - | - | - | - | - | - |
| 46 | 7 | - | - | - | - | - | - | - | - | - | - |
| 47 | 1 | - | - | - | - | - | - | - | - | - | - |
| 48 | 6 | - | - | - | - | - | - | - | - | - | - |
| 49 | 8 | - | - | - | - | - | - | - | - | - | - |
| 50 | 4 | - | - | - | - | - | - | - | - | - | - |
| 51 | 4 | - | - | - | - | - | - | - | - | - | - |
| 52 | 6 | - | - | - | - | - | - | - | - | - | - |
| 53 | 4 | - | - | - | - | - | - | - | - | - | - |
| 54 | 19 | - | - | - | - | - | - | - | - | - | - |

***Table S7:*** *Percentage of population changes in shape series.*

| From/to | **pyramid** | **plunger** | **bell** | **lower diamond** | **middle diamond** | **upper diamond** | **inverted bell** | **inverted pyramid** | **inverted plunger** | **column** | **hourglass** |
| --- | --- | --- | --- | --- | --- | --- | --- | --- | --- | --- | --- |
| 2 | 4.4% (±14.5) | 5.3% (±16.3) | -0.1% (±9.4) | -3.7% (±8.1) | -3.8% (±8.1) | -4.2% (±9.0) | -10.5% (±6.9) | -8.1% (±6.5) | -3.1% (±6.4) | -1.8% (±6.2) | 4.0% (±19.7) |
| 3 | 12.3% (±23.0) | 13.9% (±26.4) | 0.2% (±12.5) | -5.6% (±10.8) | -7.6% (±11.3) | -8.2% (±11.9) | -14.5% (±9.1) | -17.2% (±11.4) | -11.3% (±9.2) | -3.9% (±3.9) | 8.8% (±21.7) |
| 4 | 24.0% (±46.4) | 24.0% (±63.6) | -1.0% (±15.4) | -6.7% (±12.4) | -10.6% (±13.3) | -13.8% (±12.5) | -5.6% | - | -11.2% (±7.4) | -31.2% | 7.0% (±37.9) |
| 5 | 28.3% (±43.0) | 67.0% (±95.4) | 5.2% (±25.9) | -6.9% (±11.3) | -8.0% (±18.8) | -17.8% (±13.7) | - | - | -38.9% | - | 30.4% (±49.9) |
| 6 | 42.5% (±53.1) | 27.5% (±41.5) | -6.2% (±18.4) | 3.3% (±8.6) | -10.1% (±12.0) | -11.3% (±17.5) | - | - | 50.0% | - | -27.4% |
| 7 | 49.2% (±64.1) | 23.9% | 36.4% (±40.0) | 7.3% (±13.4) | -2.9% (±10.5) | -20.4% (±14.7) | - | - | - | - | -44.7% (±7.5) |
| 8 | 49.0% (±70.6) | - | 36.4% | - | - | -24.8% (±14.4) | - | -64.0% | - | - | - |
| 9 | 74.8% (±87.9) | 84.6% | - | - | - | -40.0% (±20.2) | - | - | - | - | - |
| 10 | 78.1% (±86.9) | - | - | - | - | -15.2% (±12.4) | - | - | - | - | -48.3% |
| 11 | 91.2% (±103.4) | 190.0% | - | - | - | -28.2% (±14.0) | - | - | - | - | -14.6% |
| 12 | 123.5% (±133.9) | - | - | - | - | - | - | - | - | - | - |
| 13 | 119.0% (±144.0) | - | - | - | - | -17.7% (±2.9) | - | - | - | - | - |
| 14 | 125.3% (±104.6) | - | - | - | - | -41.0% | - | - | - | - | - |
| 15 | 127.8% (±135.8) | - | - | - | - | - | - | - | - | - | - |
| 16 | 140.9% (±164.5) | - | - | - | - | - | - | - | - | - | - |
| 17 | 169.1% (±176.8) | - | - | - | - | - | - | - | - | - | - |
| 18 | 295.3% (±311.2) | - | - | - | - | - | - | - | - | - | - |
| 19 | 134.4% (±136.8) | - | - | - | - | - | - | - | - | - | - |
| 20 | 311.9% (±393.3) | - | - | - | - | - | - | - | - | - | - |
| 21 | 199.4% (±194.1) | - | - | - | - | - | - | - | - | - | - |
| 22 | 259.5% (±237.1) | - | - | - | - | - | - | - | - | - | - |
| 23 | 214.5% (±352.8) | - | - | - | - | - | - | - | - | - | - |
| 24 | 184.0% (±142.1) | - | - | - | - | - | - | - | - | - | - |
| 25 | 290.7% (±234.5) | - | - | - | - | - | - | - | - | - | - |
| 26 | 316.9% (±330.0) | - | - | - | - | - | - | - | - | - | - |
| 27 | 146.0% (±145.7) | - | - | - | - | - | - | - | - | - | - |
| 28 | 302.3% (±529.4) | - | - | - | - | - | - | - | - | - | - |
| 29 | 365.2% (±445.9) | - | - | - | - | - | - | - | - | - | - |
| 30 | 316.7% (±289.8) | - | - | - | - | - | - | - | - | - | - |
| 31 | 465.1% (±513.4) | - | - | - | - | - | - | - | - | - | - |
| 32 | 485.3% (±360.0) | - | - | - | - | - | - | - | - | - | - |
| 33 | 413.3% (±429.1) | - | - | - | - | - | - | - | - | - | - |
| 34 | 823.4% (±672.6) | - | - | - | - | - | - | - | - | - | - |
| 35 | 314.3% (±208.5) | - | - | - | - | - | - | - | - | - | - |
| 36 | 336.7% (±299.2) | - | - | - | - | - | - | - | - | - | - |
| 37 | 828.0% (±704.6) | - | - | - | - | - | - | - | - | - | - |
| 38 | 1475.6% (±2574.4) | - | - | - | - | - | - | - | - | - | - |
| 39 | 817.3% (±458.8) | - | - | - | - | - | - | - | - | - | - |
| 40 | 1109.5% (±488.0) | - | - | - | - | - | - | - | - | - | - |
| 41 | 1103.7% (±901.5) | - | - | - | - | - | - | - | - | - | - |
| 42 | 896.8% (±1257.1) | - | - | - | - | - | - | - | - | - | - |
| 43 | 981.6% (±546.9) | - | - | - | - | - | - | - | - | - | - |
| 44 | 736.2% (±721.2) | - | - | - | - | - | - | - | - | - | - |
| 46 | 1032.3% (±578.4) | - | - | - | - | - | - | - | - | - | - |
| 47 | 280.6%  (-) | - | - | - | - | - | - | - | - | - | - |
| 48 | 1177.4% (±1635.1) | - | - | - | - | - | - | - | - | - | - |
| 49 | 695.0% (±652.3) | - | - | - | - | - | - | - | - | - | - |
| 50 | 299.8% (±185.6) | - | - | - | - | - | - | - | - | - | - |
| 51 | 523.6% (±306.2) | - | - | - | - | - | - | - | - | - | - |
| 52 | 743.9% (±469.6) | - | - | - | - | - | - | - | - | - | - |
| 53 | 1309.7% (±697.1) | - | - | - | - | - | - | - | - | - | - |
| 54 | 449.7% (±385.8) | - | - | - | - | - | - | - | - | - | - |

***Table S8:*** *Classification rules based on the transitions between the n-th bucket and the (n+1)st bucket. An ‘increase’ is written as 1, a ‘decrease’ as -1 and a 0 stands for ‘no change’. We assume the ‘juvenile bucket’ to be bucket number 1 and the ‘senior bucket’ to be bucket number 5.*

| Idealised transition sequence | Additional rules | Classification |
| --- | --- | --- |
| -1, -1, 1, 1 |  | Hourglass |
| -1, -1, 1, 0 |  | Hourglass |
| -1, -1, 0, 1 |  | Hourglass |
| 1, 1, 0, 0 |  | Inverted Bell |
| 1, 0, 0, 0 |  | Inverted Bell |
| 0, 1, 0, 0 |  | Inverted Bell |
| 0, 0, -1, -1 |  | Bell |
| 0, 0, -1, 0 |  | Bell |
| 0, 0, 0, -1 |  | Bell |
| 0, 0, 1, 1 |  | Inverted Plunger |
| 0, 0, 1, 0 |  | Inverted Plunger |
| 0, 0, 0, 1 |  | Inverted Plunger |
| 1, 1, 1, 1 |  | Inverted Pyramid |
| 1, 1, 1, 0 |  | Inverted Pyramid |
| 1, 1, 0, 1 |  | Inverted Pyramid |
| 1, 0, 1, 1 |  | Inverted Pyramid |
| 0, 1, 1, 1 |  | Inverted Pyramid |
| 0, 1, 1, 0 |  | Inverted Pyramid |
| 0, 1, 0, 1 |  | Inverted Pyramid |
| 1, 0, 1, 0 |  | Inverted Pyramid |
| 1, 0, 0, 1 |  | Inverted Pyramid |
| 1, -1, -1, -1 |  | Lower Diamond |
| 1, -1, -1, 0 |  | Lower Diamond |
| 1, -1, 0, -1 |  | Lower Diamond |
| 1, -1, 0, 0 |  | Lower Diamond |
| 1, 0, -1, -1 |  | Lower Diamond |
| 1, 0, -1, 0 |  | Lower Diamond |
| 1, 1, -1, -1 |  | Diamond |
| 1, 1, -1, 0 |  | Diamond |
| 1, 0, 0, -1 |  | Diamond |
| 0, 1, -1, -1 |  | Diamond |
| 0, 1, -1, 0 |  | Diamond |
| -1, -1, 0, 0 |  | Plunger |
| -1, 0, 0, 0 |  | Plunger |
| 0, -1, 0, 0 |  | Plunger |
| -1, -1, -1, -1 |  | Pyramid |
| -1, -1, -1, 0 |  | Pyramid |
| -1, -1, 0, -1 |  | Pyramid |
| -1, 0, -1, -1 |  | Pyramid |
| -1, 0, -1, 0 |  | Pyramid |
| -1, 0, 0, -1 |  | Pyramid |
| 0, -1, -1, -1 |  | Pyramid |
| 0, -1, -1, 0 |  | Pyramid |
| 0, -1, 0, -1 |  | Pyramid |
| 1, 1, 1, -1 |  | Upper Diamond |
| 1, 1, 0, -1 |  | Upper Diamond |
| 1, 0, 1, -1 |  | Upper Diamond |
| 0, 1, 1, -1 |  | Upper Diamond |
| 0, 1, 0, -1 |  | Upper Diamond |
| 0, 0, 1, -1 |  | Upper Diamond |
| 0, 0, 0, 0 |  | Column |
| -1, -1, -1, 1 | B3 > B5  B3 <= B5 | Pyramid  Hourglass |
| 0, -1, -1, 1 | B3 > B5  B3 <= B5 | Pyramid  Hourglass |
| 1, 1, -1, 1 | B3 > B5  B3 = B5  B3 < B5 | Diamond  Inverted Bell  Inverted Pyramid |

| 0, 0, -1, 1 | B3 > B5  B3 = B5  B3 < B5 | Bell  Column  Inverted Plunger |
| --- | --- | --- |
| 0, 1, -1, 1 | B3 > B5  B3 = B5  B3 < B5 | Diamond  Inverted Bell  Inverted Pyramid |
| 1, 0, -1, 1 | B3 > B5  B3 = B5  B3 < B5 | Lower Diamond  Inverted Bell  Inverted Pyramid |
| 0, -1, 1, 0 | B2 > B4  B2 = B4  B2 < B4 | Plunger  Hourglass  Inverted Plunger |
| 0, -1, 0, 1 | B2 > B5  B2 = B5  B2 < B5 | Pyramid  Hourglass  Inverted Plunger |
| 1, -1, 1, 0 | B2 > B4  B2 = B4  B2 < B4 | Lower Diamond  Inverted Bell  Inverted Pyramid |
| 0, -1, 1, 1 | B2 > B4  B2 <= B4 | Hourglass  Inverted Plunger |
| -1, 0, 0, 1 | B2 > B5  B2 = B5  B2 < B5 | Plunger  Hourglass  Inverted Plunger |
| -1, 0, 1, 1 | B1 >= B4  B1 < B4 | Hourglass  Inverted Plunger |
| -1, 0, 1, 0 | B1 >= B4  B1 < B4 | Hourglass  Inverted Plunger |
| -1, 1, 1, 0 | B1 >= B4  B1 < B4 and B1 < B3  B1 < B4 and B1 >= B3 | Hourglass  Inverted Pyramid  Hourglass |
| -1, 1, -1, -1 | B1 > B3  B1 = B3  B1 < B3 | Pyramid  Bell  Diamond |
| -1, 1, -1, 0 | B1 > B3  B1 = B3  B1 < B3 | Pyramid  Bell  Diamond |
| -1, 1, 0, -1 | B1 > B3  B1 = B3  B1 < B3 | Pyramid  Bell  Upper Diamond |
| -1, 1, 0, 1 | B1 > B3  B1 = B3  B1 < B3 | Hourglass  Inverted Plunger  Inverted Pyramid |
| -1, 1, 1, 1 | B1 > B3  B1 = B3  B1 < B3 | Hourglass  Inverted Plunger  Inverted Pyramid |
| -1, 0, -1, 1 | B1 = B5 or B2 < B5  elseif B2 = B4  else | Hourglass  Plunger  Pyramid |
| -1, 1, 0, 0 | B1 > B3  B1 = B3  B1 < B3 | Plunger  Column  Inverted Bell |
| 1, -1, -1, 1 | B3 > B5  B3 = B5 and B1 > B3  B3 = B5 and B1 <= B3  B3 < B5 and B1 >= B3  B3 < B5 and B1 < B3 | Lower Diamond  Plunger  Lower Diamond  Hourglass  Inverted Pyramid |
| -1, 1, 1, -1 | B1 < B4  else  B1 > B3 and B3 > B5  else B3 < B5  else | Upper Diamond  Pyramid  Hourglass  Bell |
| -1, 0, 1, -1 | B1 <= B5  else  B1 > B4  B1 = B4  B1 < B4 | Upper Diamond  Pyramid  Bell  Hourglass |
| 1, -1, 1, -1 | B1 = B3 and B1 = B5  if B2 > B4  if B2 = B4  if B2 < B4  else if B1 > B3 and B3 > B5  else if B1 < B3 and B3 < B5  else if B1 > B3 and B3 < B5  B2 = B4  B2 > B4  B2 < B4  else  B2 = B4  B2 > B4  B2 < B4 | Lower Diamond  Diamond  Upper Diamond  Pyramid  Inverted Pyramid  Hourglass  Lower Diamond  Upper Diamond  Hourglass  Lower Diamond  Upper Diamond |
| 0, -1, 1, -1 | B2 > B4 and B3 > B5  B2 > B4 and B3 = B5  B2 > B4 and B3 < B5  B2 = B4 and B3 < B5  B2 = B4 and B3 >= B5  B2 < B4 | Pyramid  Plunger  Hourglass  Hourglass  Bell  Upper Diamond |
| -1, -1, 1, -1 | B1 > B4 and B3 > B5  B1 > B4 and B3 = B5  B1 > B4 and B3 < B5  B1 = B4  B1 < B4 and B1 <= B5  B1 < B4 and B1 > B5 | Pyramid  Plunger  Hourglass  Bell  Upper Diamond  Hourglass |
| 1, -1, 1, 1 | B1 > B3  B1 < B3  B1 = B3 and B2 > B4  B1 = B3 and B2 = B4  B1 = B3 and B2 < B4 | Hourglass  Inverted Pyramid  Hourglass  Inverted Pyramid  Inverted Plunger |
| -1, 1, -1, 1 | B1 > B3 and B3 > B5  B1 > B3 and B3 < B5  B1 > B3 and B3 = B5  B1 = B3 and B3 = B5  B1 = B3 and B3 > B5  B1 = B3 and B3 < B5  B1 < B3 and B3 < B5  B1 < B3 and B3 = B5  B1 < B3 and B3 > B5 | Pyramid  Hourglass  Plunger  Column  Bell  Inverted Plunger  Inverted Pyramid  Inverted Bell  Diamond |
| 1, -1, 0, 1 | B1 = B3 and B2 <= B5  B1 = B3 and B2 > B5  B1 > B3 and B2 < B5  B1 > B3 and B2 = B5  B1 > B3 and B2 > B5  B1 < B3 and B2 < B5  B1 < B3 and B2 = B5  B1 < B3 and B2 > B5 | Inverted Plunger  Lower Diamond  Hourglass  Inverted Bell  Lower Diamond  Inverted Pyramid  Inverted Bell  Lower Diamond |
